# MALDI-MSI spatially maps N-glycan alterations to histologically distinct pulmonary pathologies following irradiation

**DOI:** 10.1038/s41598-020-68508-y

**Published:** 2020-07-14

**Authors:** Claire L. Carter, George A. Parker, Kim G. Hankey, Ann M. Farese, Thomas J. MacVittie, Maureen A. Kane

**Affiliations:** 1 Department of Pharmaceutical Sciences, School of Pharmacy, University of Maryland, Baltimore, MD USA; 2 Department of Radiation Oncology, School of Medicine, University of Maryland, Maryland, USA; 3 Pathology Associates, Charles River Laboratories, Raleigh-Durham, NC USA

**Keywords:** Imaging, Mass spectrometry

## Abstract

Radiation-induced lung injury is a highly complex combination of pathological alterations that develop over time and severity of disease development is dose-dependent. Following exposures to lethal doses of irradiation, morbidity and mortality can occur due to a combination of edema, pneumonitis and fibrosis. Protein glycosylation has essential roles in a plethora of biological and immunological processes. Alterations in glycosylation profiles have been detected in diseases ranging from infection, inflammation and cancer. We utilized mass spectrometry imaging to spatially map N-glycans to distinct pathological alterations during the clinically latent period and at 180 days post-exposure to irradiation. Results identified alterations in a number of high mannose, hybrid and complex N-glycans that were localized to regions of mucus and alveolar-bronchiolar hyperplasia, proliferations of type 2 epithelial cells, accumulations of macrophages, edema and fibrosis. The glycosylation profiles indicate most alterations occur prior to the onset of clinical symptoms as a result of pathological manifestations. Alterations in five N-glycans were identified as a function of time post-exposure. Understanding the functional roles N-glycans play in the development of these pathologies, particularly in the accumulation of macrophages and their phenotype, may lead to new therapeutic avenues for the treatment of radiation-induced lung injury.

## Introduction

Radiation-induced lung injury (RILI) is a complex combination of pathologies that include vascular damage, epithelial cell death followed by hyper-proliferation, edema, and mixed immune infiltrations resulting in pneumonitis and an aberrant wound-healing processes that ultimately lead to the development of pulmonary fibrosis^1–4^. Accidental or intentional exposures to high doses of ionizing radiation results in RILI, which contributes to increased risk of mortality within months^3,4^. RILI is also a common side effect of thoracic radiotherapy for treatment of malignancies; it often limits curative therapeutic strategies^1,5^. Whereas RILI due to radiotherapy can be controlled to prevent mortality, accidental or intentional exposures to lethal doses of ionizing radiation result in significant lung disease development^3,6–8^. To date there are no Food and Drug Administration (FDA) approved therapeutic options to treat or mitigate RILI and the molecular mechanisms leading to the development of pulmonary fibrosis remain incompletely understood.

Protein glycosylation is a post-translational modification that plays essential biological roles in structural and modulatory processes, and cell–cell recognition^9^. More specifically, glycoproteins are involved in protein folding, molecular trafficking and clearance, and secretion processes^10–12^. On the cell surface, membrane-bound glycoproteins are binding ligands for host-host interactions that include cell adhesion, receptor activation and extracellular matrix molecules, and host–pathogen interactions that enable bacteria and viruses to attach and gain entry into mammalian cells^9,13,14^. Glycosylation of proteins and lipids are central to many regulatory processes of the innate and adaptive immune system including cell trafficking, cell proliferation and differentiation, T and B cell receptor function, antigen presentation and antibody function and clearance^15–17^. Protein glycosylation is an enzymatic process that attaches oligosaccharides to proteins as N-linked or O-linked glycans. O-glycans are covalently attached to the oxygen of the hydroxyl group of serine, threonine or tyrosine^18^. N-glycans are attached via the nitrogen of the amide side chain of an asparagine residue in the consensus sequence Asn-X-Ser/Thr, where X can be any amino acid except proline^19,20^. N-glycan structure can be readily probed by mass spectrometry following enzymatic cleavage using the endoglycosidase, PNGase F^21^. N-glycan structure is based on a common core composition of N-acetylglucosamine (GlcNAc) and mannose (Man) residues in the following sequence, Manα1–6(Manα1–3)Manβ1–4GlcNAcβ1–4GlcNAcβ1-Asn-X-Ser/Thr^22^. This core structure is then diversified by the addition of several sugar residues including, mannose, glucose, galactose, N-acetylglucosamine, N-acetylgalactosamine, fucose and N-acetylneuraminic acid. The order of addition of these compositions gives rise to three distinct structures known as high mannose, hybrid and complex glycans^22^. There is, however, huge diversity that exists within these categories due to the number of additions and their combinations. Changes in glycoprotein function, due to variations in the linkage type, monosaccharide units, and branching, alters cellular phenotypes and are known to be involved in numerous disease states^9,23,24^.

Matrix-assisted laser desorption ionization mass spectrometry imaging (MALDI-MSI) is a novel tool that can be used to probe molecular alterations (proteins, lipids, metabolites and glycans) and spatially map the alterations to regions within the pulmonary microenvironment^25–28^. MALDI-MSI has previously identified lipidomic dysregulation relating to surfactant abnormalities, inflammation and fibrosis during the later stages of RILI following exposure to lethal doses of ionizing radiation^29^. MALDI-MSI has also been used to map the spatial distribution of a potential medical countermeasure, AEOL10150, during efficacy studies^30^. Recent studies have utilized MALDI-MSI to delineate changes in glycosylation patterns at the spatial level between tumor, stroma and normal tissue for a number of cancers, including ovarian, hepatic and prostate^24,25,31–35^. To date, no studies have examined the spatial locality of N-glycans in lung tissue or the alterations thereof that occur during the development of RILI.

This current study utilizes MALDI-MSI and histopathology to spatially map N-linked glycan alterations during the clinically latent stage and the later, clinically symptomatic stages of RILI. Glycan alterations were localized to regions of mucus accumulation, edema, macrophage accumulations, epithelial cell hyperplasia and fibrosis.

## Results

### Clinically latent period: histology at 50 days post-exposure

**Figure 1:**
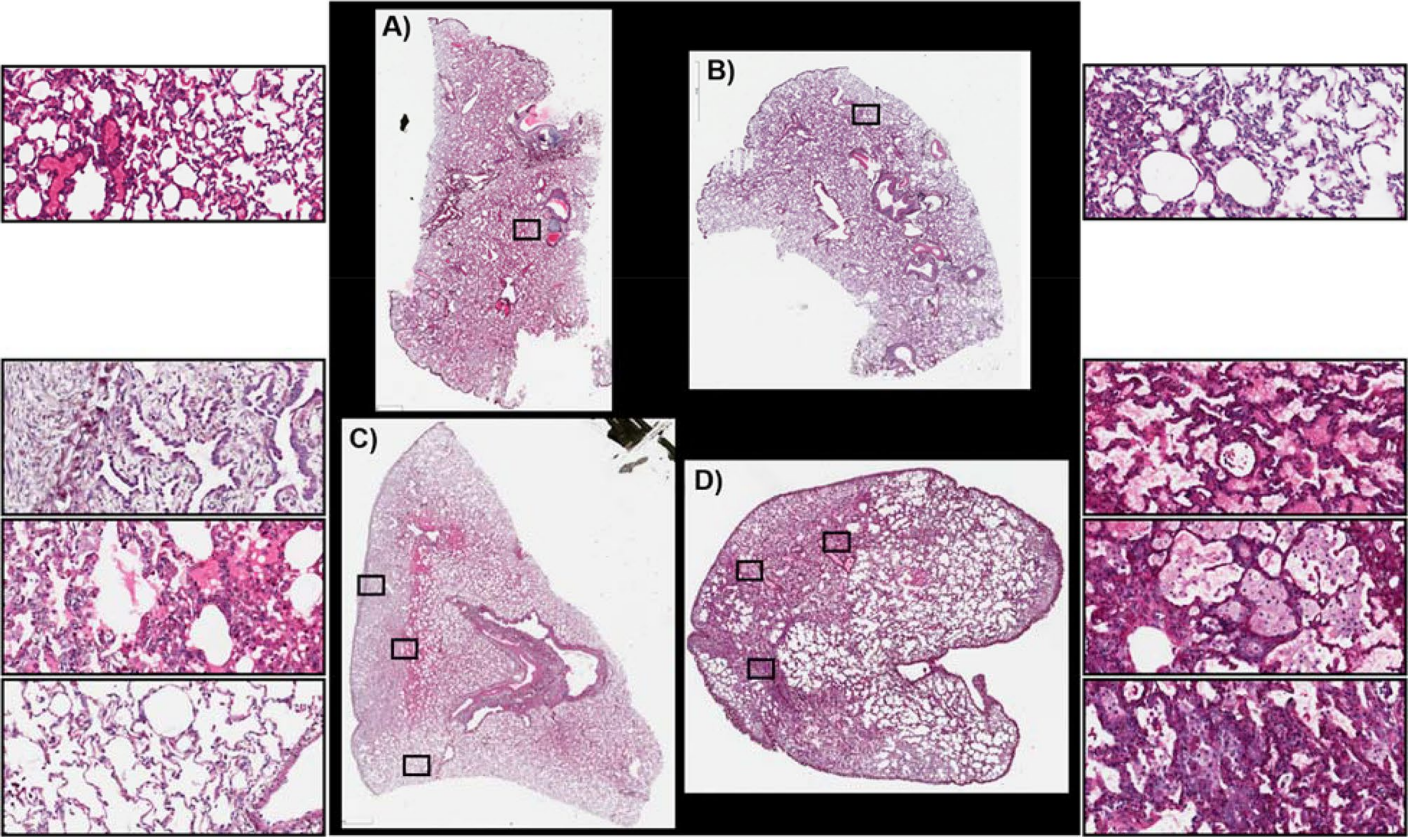
Histology at 50 days post-exposure. Hematoxylin and eosin stained sections of control (**A**, 0879 and **B**, 0147) and IR animals (**C**, 040205 and **D**, 061877). Higher magnification of regions of lung tissue are represented by the boxes.

The clinically latent period occurs within the first two months post-exposure. During this time molecular and histological changes are occurring but there remains enough functioning lung capacity that no clinical symptoms are observed. All non-human primates (NHP) analyzed during the clinically latent period experienced the coincident development of the GI- and H-ARS plus kidney and prolonged GI damage^4,36–42^. Euthanasia during this period was due to the manifestation of these injuries and not due to respiratory distress. Histopathological characterization of the development of RILI over the time-course of 180 days in this PBI model has recently been published^4^. The samples used for MSI interrogation were taken from the same study and are representative of the pathological alterations observed in this model. Histological images of the stained sections used for MSI analysis, taken from control and irradiated (IR) animals during the clinically latent period, are shown in Fig. 1A–D and Supplementary Figs. S1–S3. Both control sections show regions of collapsed lung and regions of inflated airways, Fig. 1A,B. Regions of collapsed lung architecture is a common occurrence in lung sections that have not been manually or artificially inflated during sample preparation^43^. The section taken from IR animal 040205 is shown in Fig. 1C with higher magnification regions shown in the boxes to the left and in Supplementary Fig. S2A–C. The lung section from this animal displayed regions of immature pleural and subpleural interstitial fibrosis (top box), regions of extensive alveolar edema (middle box), and regions of normal appearing airways and alveoli (bottom box). A lung section from a second IR animal, 061877, shows regions with multiple different pathological alterations, Fig. 1D and Supplementary Fig. S3A–E. The presence of alveolar edema is highlighted by the accumulation of eosinophilic (pink) fluid protein material (top box). Alveolar macrophage accumulation and interstitial thickening can be observed in the middle box and a mixture of alveolar macrophage accumulation and proliferations of type 2 alveolar epithelial cells (AEC2) is shown in the bottom box. More detailed labeling and higher magnification images of these alterations along with example Masson’s trichrome stains from the same lung samples are shown in Supplementary Figs. S2A–C and S3A–E. Whereas both sections had regions of interstitial thickening and fibrosis, the levels of fibrosis in each lung varied as evidenced by the blue collagen staining in the trichrome sections presented in Supplementary Figs. S2A–C and S3A–E. The pleural and interstitial fibrosis in regions of IR sample 040205 were predominantly loose connective tissue of non-organized collagen bundles mixed with other components of extracellular matrix. This is typical of early manifestations of fibrosis. Whereas the fibrosis in IR sample 061877 was detected throughout the section and was more organized and tightly packed as evidenced by comparing the blue staining intensity in the trichrome stains presented for each sample in Supplementary Figs. S2A and S3A–E.

**Figure 2:**
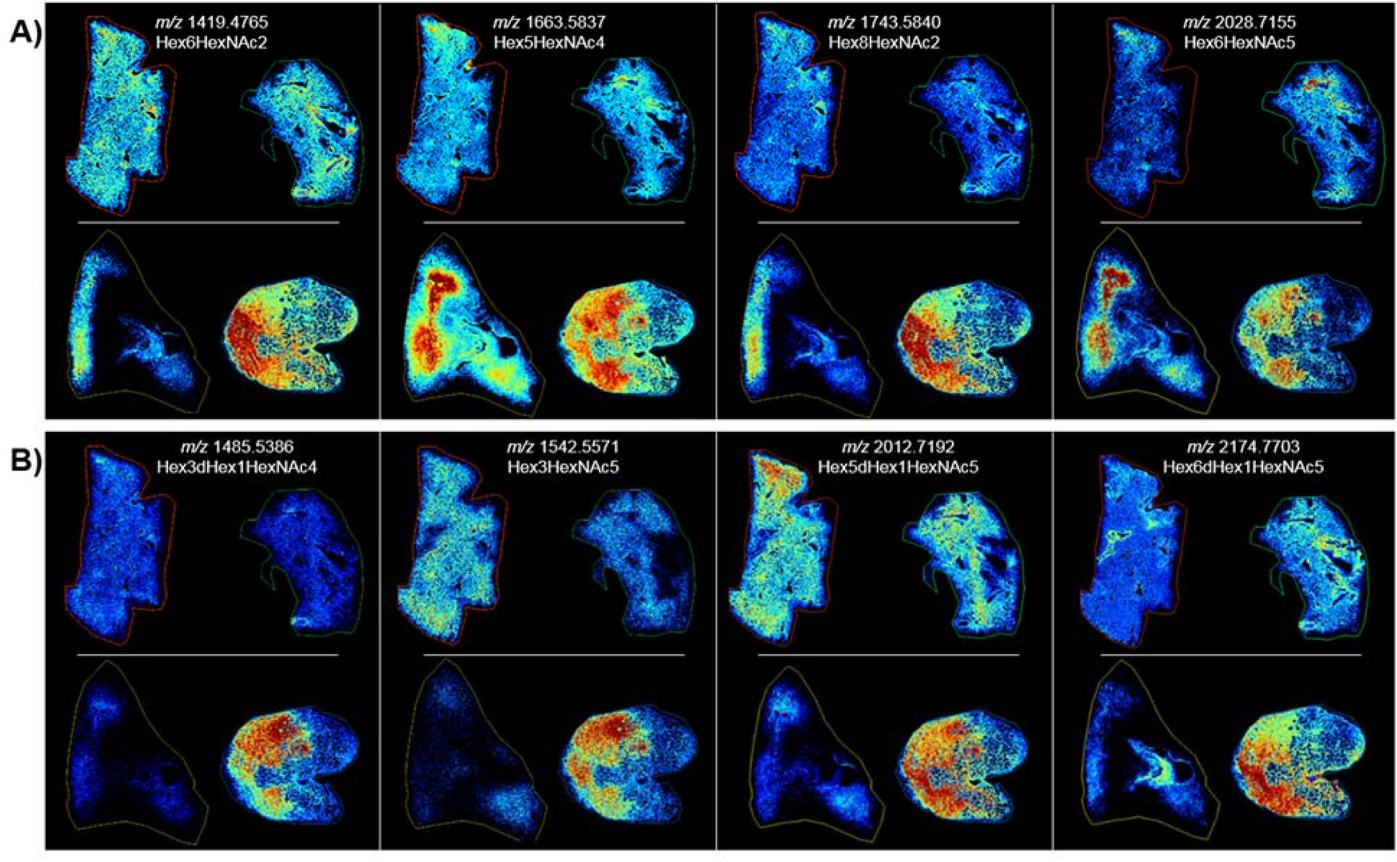
MSI at 50 days post-exposure. MALDI-MS images of N-linked glycans taken from control (top images of** A** and** B**, L–R, 0879 and 0147) and 50 days ± 10 post-irradiation (bottom images of **A** and **B**, L–R, 040205 and 061877). Glycan structures were detected as the [M + Na] + ion and tentatively assigned. Hex = Hexose, dHex = Fucose and HexNAc = N-acetylhexosamine.

**Figure 3:**
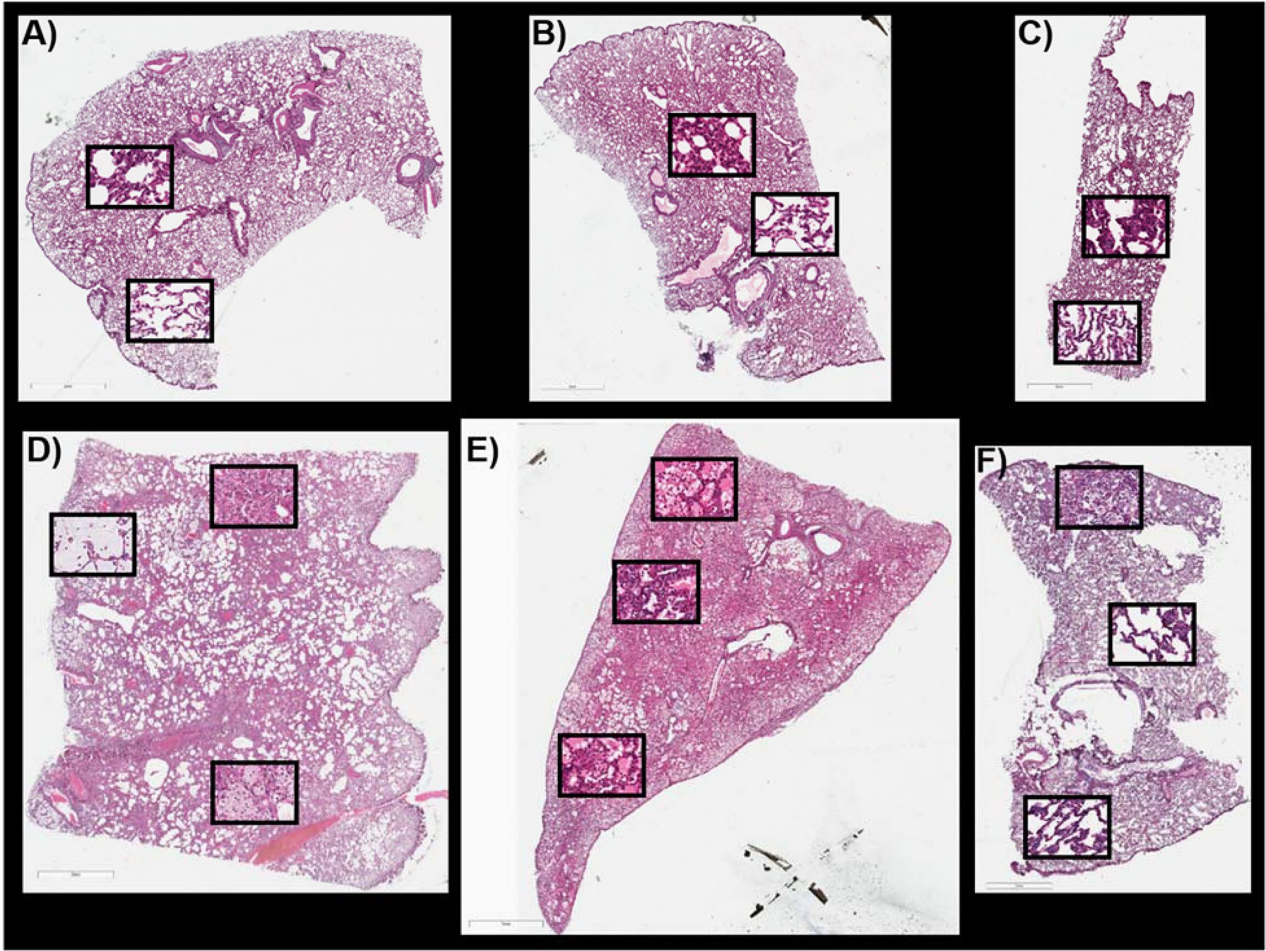
Histology at 180 days post-exposure. Hematoxylin and eosin stained sections of control (**A**-0147, **B**-0879 and **C**-08094011) and IR animals (**D**-04923, **E**-R03007 and **F**-040087). Higher magnification of regions of lung tissue are shown in the boxes.

### Clinically latent period: glycan alterations detected by MSI at 50 days post-exposure

A number of N-linked glycans displayed an increased intensity that were localized to microscopically distinct pathologies during the clinically latent period following radiation insult. Increases in high mannose structures across both IR samples were localized to regions of fibrosis, as shown by the example data presented in Fig. 2A for Hex6HexNAc2 and Hex8HexNAc2. Increased intensity in these glycans along with the high mannose structures, Hex7HexNAc2 and Hex9HexNAc2, were detected within the regions that displayed pleural and interstitial fibrosis in IR sample 040205 and regions of interstitial fibrosis and alveolar macrophage accumulation in IR sample 061877. There was also a high degree of AEC2 and alveolar-bronchiolar hyperplasia within these regions of 061877 as demonstrated by comparing the MS images of these glycans to the histology images presented in Fig. 1 and Supplementary Figs. S2 and S3. The difference in intensity between the two IR samples is attributed to differences in pathology. The pleural and interstitial fibrosis in the section from 040205 is mostly loose connective tissue that consists of non-organized collagen bundles mixed with other components of extracellular matrix. This is typical of early manifestations of fibrosis and thus the collagen stain is very faint in the trichrome section (Supplementary Fig. S2A). Conversely, the fibrosis in section 061877 is more organized and densely packed, and thus stains dark blue on the trichrome stain (Supplementary Fig. S3C–E). Several glycan compositions were detected that demonstrated increased intensity in regions of edema in both IR samples, with slightly lower intensity in the fibrotic regions. Examples of this are shown in Fig. 2A for the hybrid/complex glycans, Hex5HexNAc4 and Hex6HexNAc5. The addition of core fucosylation to Hex5HexNAc4, giving a composition of Hex5HexNAc4dHex1, resulted in a slightly differential distribution. This glycan demonstrates a higher distribution in vessel walls and regions of fibroconnective tissue surrounding bronchi in control tissue and IR sample 040205, and increased detection that is more localized to the fibrotic alveolar regions in the IR sample 061877 (Supplementary Fig. S4, middle panel). Examination of several other hybrid/complex glycans with and without core fucosylation also show increased detection in vessels and collagen rich structures in control and IR lung sections; however, these glycans only displayed an increased signal in regions of lung taken from 061877 as demonstrated by the images for the glycans, Hex4HexNAc4 and Hex4HexNAc4dHex1, and Hex5HexNAc4NeuAc1 and Hex5HexNAc4NeuAc1dHex1 in Supplementary Fig. S4. The differences in glycan signal intensity between the two IR samples is attributed to the differences in the observed histopathology and thus the cellular and molecular content of the samples. Specifically, these glycans are increased in the regions of mucus and macrophage accumulations, fibrosis and severe AEC2 hyperplasia that is present in 061877 but not in 040205. These differences are notable upon comparison of the MSI figures with the higher magnification figures of the histologically stained regions in Supplementary Figs. S2 and S3.

Several more N-glycans were detected with an increased intensity in only IR sample 061877 and these differences are again attributed to differences in pathology. Two glycans displayed increased intensity in regions that contained mucus accumulation in the alveolar space, followed by lower signal intensity in regions of macrophage accumulation, both regions contained interstitial fibrosis. These can be observed by comparing the MS images of Hex3HexNAc5 and Hex3HexNAc5dHex1 in Fig. 2B with the higher magnification regions of these areas in the histology sections presented in Supplementary Fig. S3A, B. Three glycans were detected with increased intensity across all pathologies in 061877, as shown by the third panel in Fig. 2B and Supplementary Fig. S5 for Hex3HexNAc5dHex1, Hex4HexNAc5dHex1 and Hex5HexNAc5dHex1. These glycans were all detected with highest intensity in the parenchyma of the control tissue with low signal in regions of fibroconnective tissue surrounding the vessels and bronchi. As the signal is low in regions of connective tissue in control sections, the increases observed for these glycans in the parenchyma of the IR sample is not believed to be due to excess collagen deposition, but more to do with differences in the cells and/or cell populations. The last glycan presented in Fig. 2B, Hex6HexNAc5dHex1, was detected in IR sample 061877 with increased intensity in regions of fibrosis and AEC2 hyperplasia. Representative mass spectra from the lung parenchyma of control sections and the regions of differing pathology from the parenchyma of the two IR samples is shown in Supplementary Fig. S7.

A decrease in intensity for the high mannose structure Hex5HexNAc2 and the hybrid/complex glycan, Hex5HexNAc5dhex2 was detected across both IR samples compared to the control samples, with a more pronounced signal reduction observed in IR sample 040205 compared to IR sample 061877 (Supplementary Fig. S6). This is again thought to be due to differences in cellular and protein complexity between the samples as 061877 presented with more severe pathology and thus a greater number of cellular and molecular content. 040205 also had regions of normal appearing pulmonary architecture that maintained its physiologically inflated state (Supplementary Fig. S2C). Meaning many regions were just the alveolar wall and thus the apparent decrease in intensity in these areas correlates with less sample material being ablated compared to the collapsed control tissue and the diseased IR samples. Intensity boxplots for several glycans detected within the different pathological presentations are shown in Supplementary Figs. S15, S16 and data correlates with those presented in the MSI figures.

### End of study: histology at 180 days post-exposure

Histological images of the sections used for MSI, from control and IR animals at 180 days post-exposure, are shown in Fig. 3A–F and Supplementary Figs. S8–S10. The first IR section, taken from the IR animal designated 04298, displayed multiple regions of mucus accumulation (top left box, Fig. 3D), alveolar macrophage accumulations (bottom box, Fig. 3D), and alveolar-bronchiolar hyperplasia (top center box, Fig. 3D). AEC2 hyperplasia and fibrosis was observed throughout this section. More detailed microscopic labeling and higher magnification images for these pathologies are shown in Supplementary Fig. S8A–E. The second IR lung section taken from the IR animal designated R03007, had regions of extensive alveolar edema throughout the section, as evidenced by the eosinophilic (pink) protein content that fills the alveolar spaces. There was also extensive mixed immune cell infiltration that appeared to be predominantly lymphocyte populations, top box Fig. 3E. Regions of edema, fibrosis, AEC2 and alveolar-bronchiolar hyperplasia (middle box, Fig. 3E) and regions of edema with macrophage accumulations in the alveolar space (bottom box, Fig. 3E) are present. This lung section was the most severely affected of the three and fibrosis was detected throughout the section. More detailed microscopic labeling and higher magnification images for IR sample R03007 are shown in Supplementary Fig. S9A–E, along with Masson’s trichrome staining to demonstrate the degree of collagen deposition for fibrosis severity in this sample. The third lung section taken from IR animal 040087 at 180 days post exposure showed regions of AEC2 and alveolar-bronchiolar hyperplasia, alveolar macrophage accumulation and dense subpleural alveolar fibrosis (top box, Fig. 3F) and regions of normal appearing lung parenchyma (middle and bottom boxes, Fig. 3F). More detailed pathology labeling and higher magnification images for this section is shown in Supplementary Fig. S10A–C, along with trichrome staining to demonstrate the degree of collagen deposition for fibrosis severity. This lung displayed the least severe pathological alterations and as the changes were similar to the regions in the other IR sections, the MSI data from this section will not be discussed in detail.

### End of study: glycan alterations detected at 180 days post-exposure

**Figure 4:**
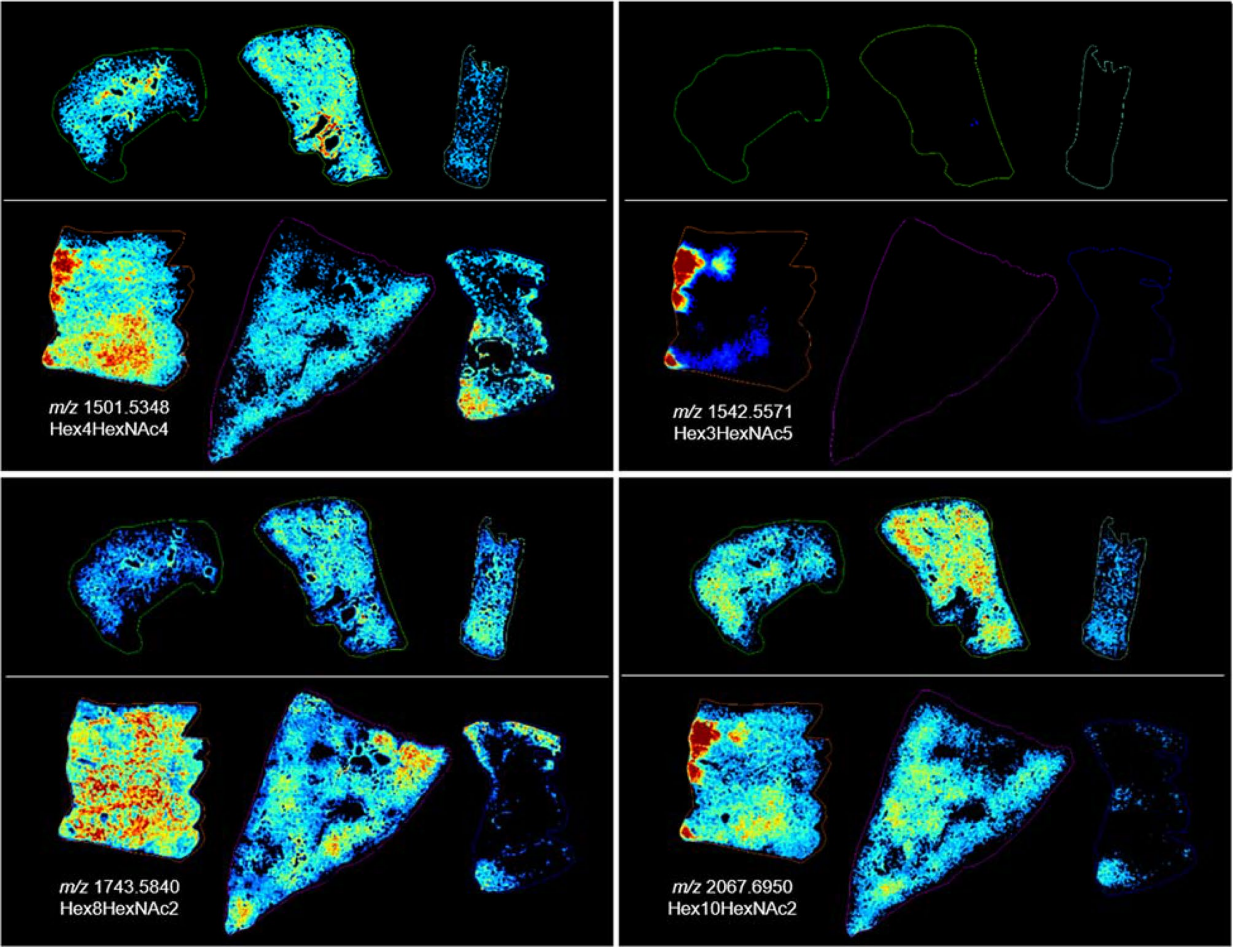
Histology at 180 days post-exposure. Glycan images showing increases in relative intensity specific to distinct pathologies in the IR lungs (bottom images in each panel) compared to the control samples (top images in each panel). Top images in each panel are control, animal ID numbers as shown left to right: 0147, 0879, 08094011. Bottom images in each panel are IR, animal ID numbers as shown left to right: 04923, R03007, 040087. Changes were more pronounced in the sample from 04923, bottom left of each panel. Glycan structures were detected as the [M + Na] + ion and tentatively assigned. Hex = Hexose, dHex = fucose and HexNAc = N-acetylhexosamine.

**Figure 5:**
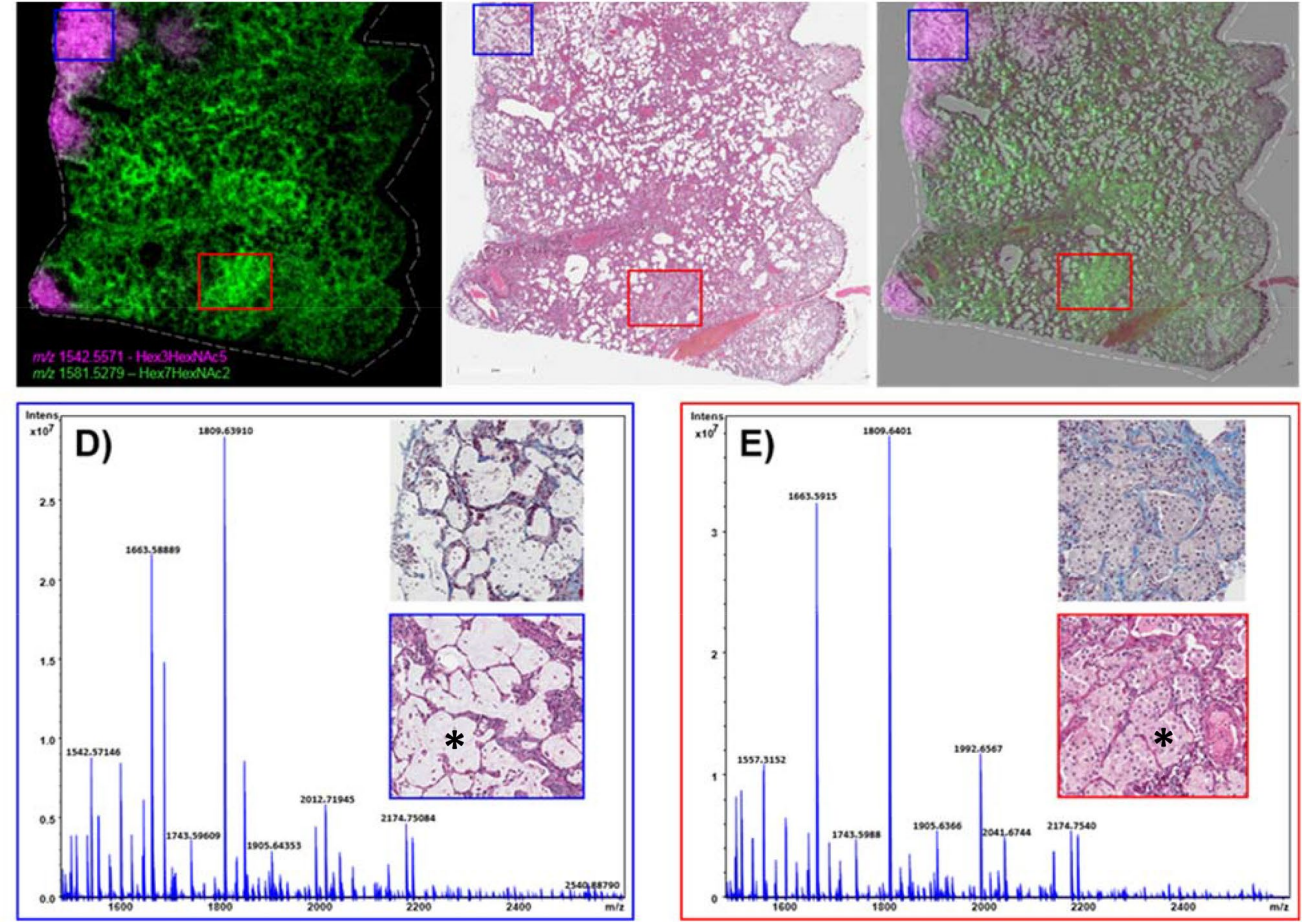
MSI and mass spectra at 180 days post-exposure. MALDI-MS merged image of two high mannose glycan structures, tentatively assigned as Hex3HexNac5 and Hex7HexNac2, showing differential distribution in a lung sample taken at 180 days post-radiation (from 04923) in (**A**). The H&E stained section is shown in (**B**) and the MSI overlaid with the H&E section is shown in (**C**). Single pixel spectra and higher magnification stained sections are shown for regions of mucin accumulation (**D**) and blue boxes, and regions of high accumulation of alveolar macrophages (**E**) and red boxes.

MALDI-MSI analysis of lung samples taken at the end of the study, day 180 post-IR, revealed differences in the distribution of N-glycan structures that correlated with the pathological presentation for each section analyzed. Correlation of the distribution of glycan ions with their histological images following radiation insult also enabled a more detailed understanding of the physiological distribution of N-glycans in control lung sections. For example, a number of glycans were detected with high intensity in regions of mucus accumulation within the alveolar space in section 04923, as demonstrated by comparing the MS images presented in Fig. 4 and SF11 to the H&E image presented in Fig. 3 and Supplementary Fig. S8A. The glycans, Hex3HexNAc4dHex1, Hex4HexNAc4, Hex3HexNAc5, Hex3HexNAc5dHex1, Hex4HexNAc5dHex1 and the high mannose glycan, Hex10HexNAc2 were all increased in regions that correlated to mucus accumulation in sample 04923. Hex4HexNAc4, Hex3HexNAc4dHex1 and Hex3HexNAc5dHex1 were also detected with increased intensity in regions of alveolar macrophage accumulation in this sample as can be observed when comparing the MS images to the histology images presented in Supplementary Fig. S8B and C. It should be noted that this region also contained AEC2 hyperplasia and/or alveolar bronchiolar hyperplasia and it is highly likely that all cell populations are contributing to the signal increase observed for these glycans. Several high mannose glycans demonstrated increased intensity in regions of interstitial fibrosis, alveolar macrophage accumulation and regions of alveolar proliferation that was more prominent in sample, 04923 as shown in the bottom left panel of Fig. 4 and Supplementary Fig. S12 for glycans Hex8HexNAc2, Hex6HexNAc2, Hex7HexNAc2. A merged ion image of the mucus associated glycan, Hex3HexNAc5, and the high mannose glycan, Hex7HexNAc2 that is increased in regions of alveolar macrophage accumulation, AEC2 hyperplasia and interstitial fibrosis is shown in Fig. 5A. Its H&E stained section is shown in Fig. 5B, and the MSI and H&E overlaid image is shown in Fig. 5C. An example of how pathology-specific glycan alterations are detected is shown in the MSI and H&E overlaid image and the extracted single pixel mass spectra taken from these regions (Fig. 5D,E). The spectrum in Fig. 5D was taken from the region of mucus accumulation highlighted by the blue box, the specific location is shown (*) in the higher magnification image in the spectrum, note the increase in the ion at *m/z* 1542.5715 for Hex3HexNAc5 compared to the spectra from control parenchyma presented in Supplementary Fig. S7, and the IR spectra taken from different pathological presentations. The spectrum exported from regions of alveolar macrophage accumulation was taken from the region shown in the red box in Fig. 5A–C and the higher magnification H&E showing the accumulation of macrophages in the alveolar space is shown inset of the spectrum in Fig. 5E. Again, the precise location the spectrum was taken from is shown (*). The example spectrum demonstrates alveolar macrophage specific glycans that share similar spectral profiles to those detected from the parenchyma of control tissue, but different ratios are present, as demonstrated in the MSI figures throughout this manuscript. For example, there is a reduction in Hex4HexNAc5dHex1 at *m/z* 1850.6519, this is evident by comparing the spectrum from this region to the control spectra and the spectra taken from the differing pathological regions of the IR samples presented in Fig. 5 and Supplementary Fig. S7, and by the image of this glycan presented in Supplementary Fig. S11.

**Figure 6:**
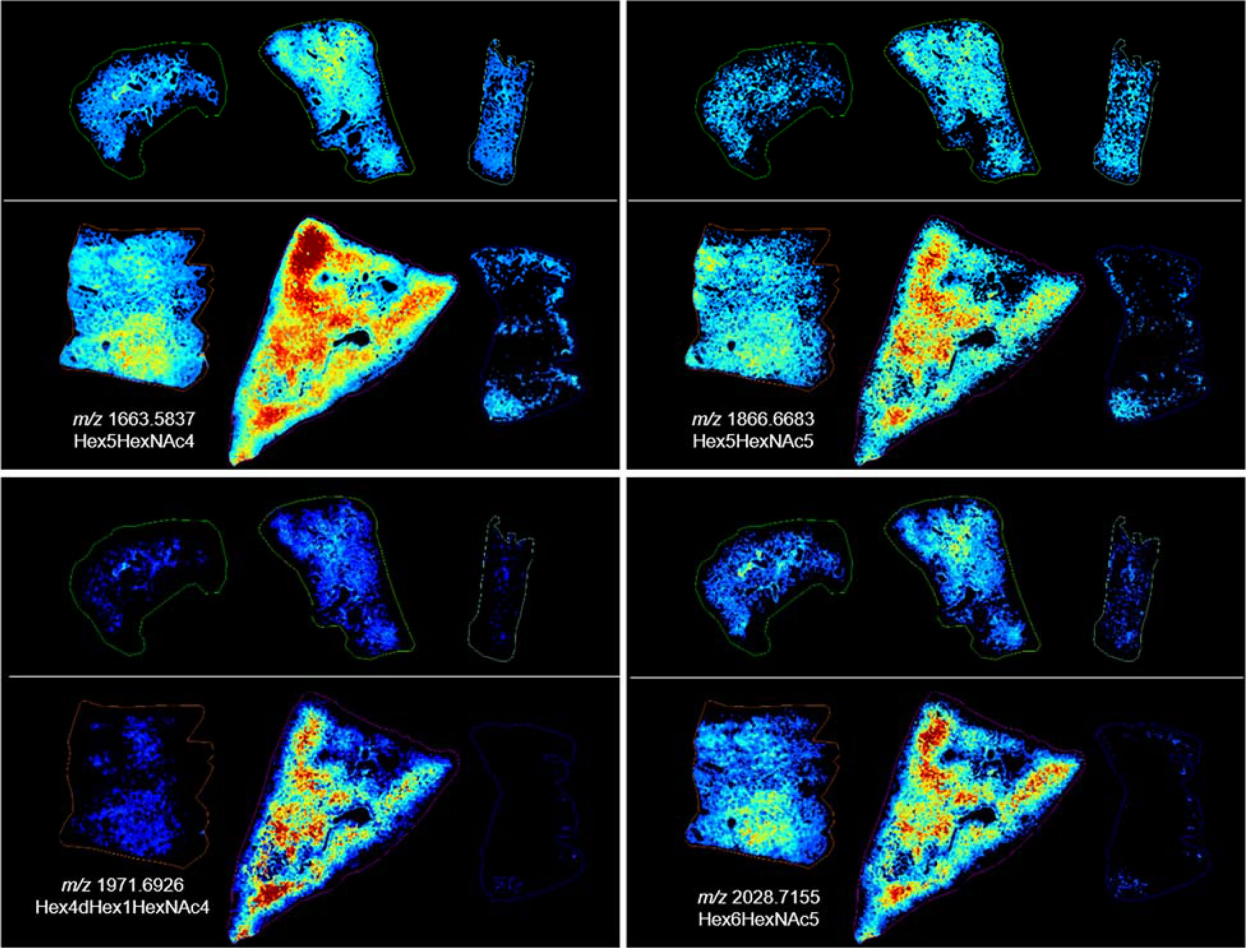
MSI at 180 days post-exposure. Glycan images showing increases in relative intensity specific to distinct pathologies in the IR lung from R03007 (bottom center image in each panel) compared to the control (top images in each panel) and other IR samples (on bottom left and right). Top images in each panel are control, animal ID numbers as shown left to right: 0147, 0879, 08094011. Bottom images in each panel are IR, animal ID numbers as shown left to right: 04923, R03007, 040087. Glycan structures were detected as the [M + Na]^+^ ion and tentatively assigned. Hex = Hexose, dHex = fucose and HexNAc = N-acetylhexosamine.

**Figure 7:**
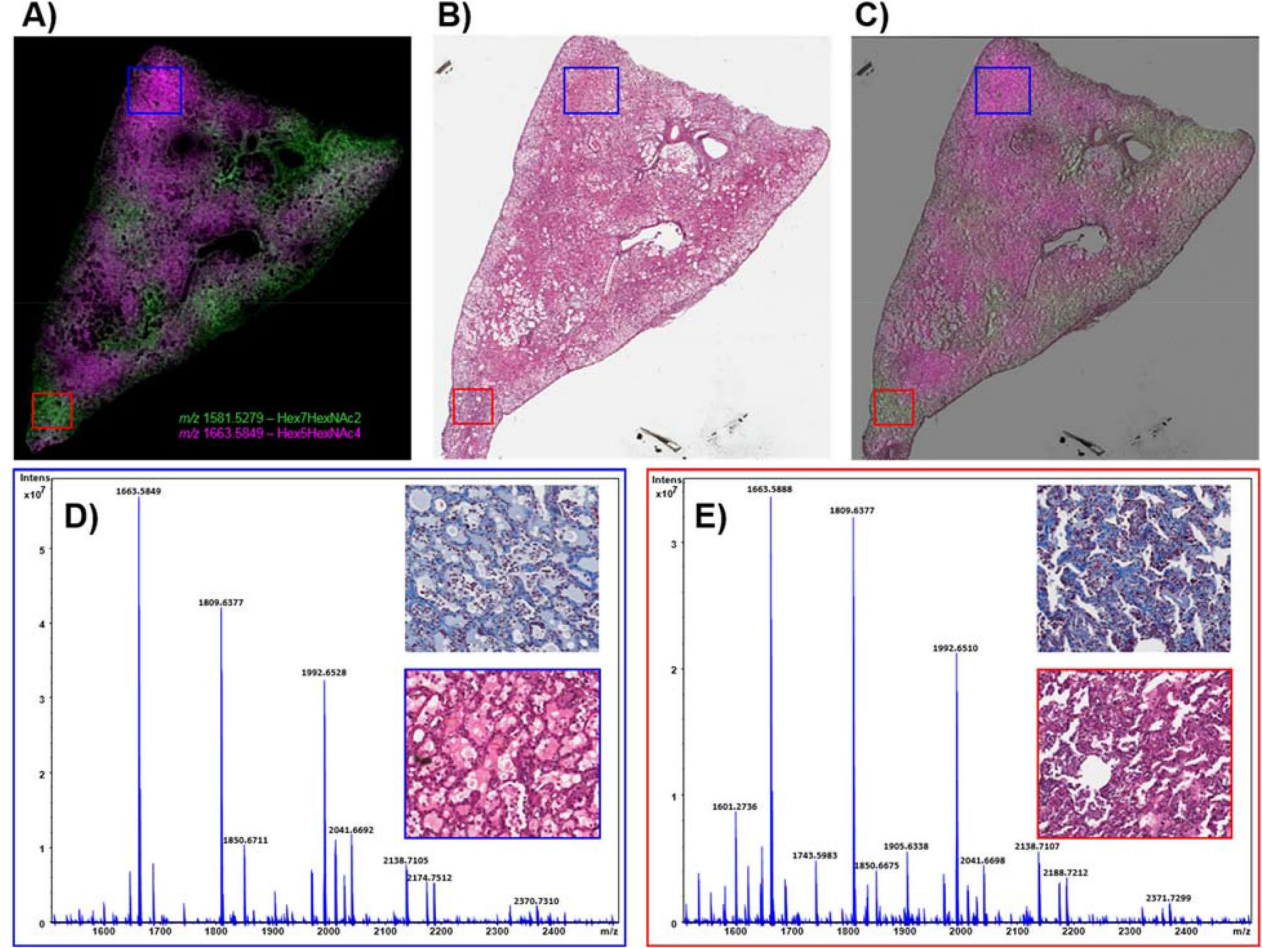
MSI and mass spectra at 180 days post-exposure. MALDI-MS merged image of two high mannose glycan structures, tentatively assigned as Hex5HexNac4 and Hex7HexNac2, showing differential distribution in a lung sample taken at 180 days post-radiation (from R03007) in (**A**). The H&E stained section is shown in (**B**) and the MSI overlaid with the H&E section is shown in (**C**). Single pixel spectra and higher magnification stained sections are shown for regions of edema and mixed immune infiltration (**D** and blue boxes), and regions of interstitial fibrosis and immune infiltration (**E** and red boxes).

The high mannose structures that displayed a more intense distribution in sample 04923 were often detected with regional increases in sample R03007 as shown in the bottom left panel of Fig. 4 and Supplementary Fig. S12. This sample also demonstrated regions of macrophage accumulation (Supplementary Fig. S9B), alveolar hyperplasia (Supplementary Fig. S9E) and both samples stained blue for increased collagen deposition throughout the lung. The collagen deposition in R03007 appeared more severe as the staining was more intense in the interstitium in this sample. For this reason, the regional increases in these glycans appear to indicate they are coming from infiltrating cellular/protein content rather than the fibrotic interstitium, as the increases are focal and not throughout the section. While both lungs presented with levels of fibrosis, alveolar macrophage accumulation, AEC2 and alveolar-bronchiolar hyperplasia, R03007 also had severe edema and mixed immune cell infiltrations that were not observed in the other lung sections taken at 180 days post-exposure. Several glycans were detected with increased intensity in these regions of this lung only, as shown in Fig. 6 and Supplementary Fig. S13 for, Hex5HexNAc4, Hex5HexNAc5, Hex4HexNAc5dHex2, Hex4HexNAc4dHex1, Hex6HexNAc5, Hex5HexNAc4NeuAc1, Hex6HexNAc5NeuAc1 and Hex7HexNAc6. These glycans were all increased in regions of R03007 that had high levels of edema and mixed immune cell accumulations that were more predominantly lymphocytes. Hex7HeNAc6 was also increased in regions of alveolar macrophage accumulation in 04923. Overlaying MSI data (Fig. 7A) with its H&E stained section (Fig. 7B, overlay in Fig. 7C) for R03007 again enabled the identification of pathology specific N-glycans as demonstrated by the extracted single pixel spectra in Figs. 7D for edema and 7E for interstitial fibrosis and mixed immune cell infiltrations. The merged ion image in Fig. 7A displays Hex5HexNAc4 a glycan that was detected with increased intensity in all regions of edema in both the end of study and latent samples, and the high mannose glycan, Hex7HexNAc2, was detected predominantly in regions of fibrosis, AEC2 hyperplasia and macrophage accumulation. The H&E section (Fig. 7B) and the MSI overlaid with H&E provides further evidence for the pathology specific distribution of these glycans. Higher magnification regions of these pathologies along with correlating trichrome stain for collagen are shown inset of each mass spectrum. There is a complete shift in the ratios of glycans compared to control samples as evidenced by comparing these spectra to the spectra presented in Supplementary Fig. S7. The biggest differences are observed for Hex5HexNAc4 at *m/*z 1663.58 and its sialic acid derivative Hex5HexNAc4NeuAc1 at *m/z* 1992.65. Intensity boxplots for several glycans detected within the different pathological presentations are shown in Supplementary Figs. S17, S18 and data correlates with those presented in the MSI figures.

A number of N-glycans were detected with decreased intensity in the IR lung sections compared to the control sections irrespective of the pathological presentation. The N-glycans, Hex5HexNAc2, Hex5HexNAc4dHex2, Hex5HexNAc5dHex2, Hex6HexNAc6dHex1, Hex5HexNAc6dHex1, Hex2HexNAc7NeuAc2, and Hex6HexNAc5NeuAc1 were all detected with decreased intensity as demonstrated in SF14.

### Glycan correlations in IR samples as a function of time post-exposure

**Figure 8:**
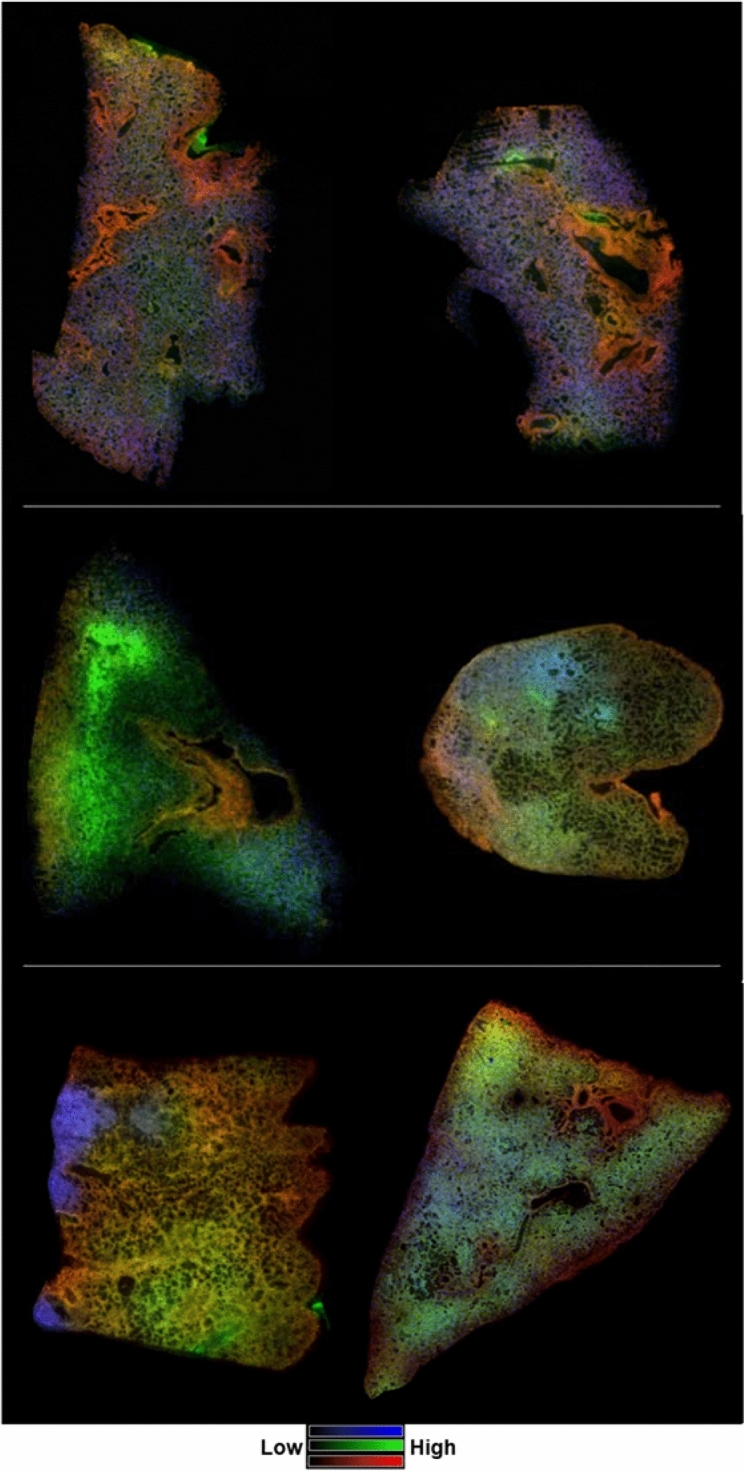
MSI of select glycans as a function of time post-exposure. Merged ion images of 3 N-linked glycans taken from control (top images, L–R, 0879 and 0147), 50 days post-irradiation (middle images, L–R, 040205 and 061877) and 180 days post-exposure (bottom images, L–R, 04923 and R03007). Glycan structures displayed are; Hex3HexNAc5 at *m/z* 1542.5571 in blue; Hex5HexNAc4 at *m/z* 1663.5837 in green; Hex5HexNAc4dHex1 at *m/z* 1809.6329 in red. Hex = Hexose, dHex = Fucose and HexNAc = N-acetylhexosamine.

By comparing the glycan alterations during the clinically latent period to the end of study a number of similarities and differences can be observed that are related to pathological presentation and time-post exposure. For example, the identification of N-glycans that were detected with high intensity in regions of mucus and alveolar-bronchiolar hyperplasia, were consistent with dysregulated mucus secretion following radiation insult at all time-points examined post-exposure. This is more easily observed in the merged ion images presented in Fig. 8. The mucus-associated glycan Hex3HexNAc5 (shown in blue, Fig. 8) displayed an even distribution in the parenchyma of control tissues. This homogenous distribution is not observed in the IR tissue samples regardless of the time post-exposure.

There are regional accumulations of Hex3HexNAc5 in the IR samples that correlate to localized increases in mucus accumulation due to alveolar-bronchiolar hyperplasia (061877 middle right panel and 04923, bottom left panel) or regions of normal appearing architecture (040205, middle left panel). The reduction in mucus-associated N-glycans correlated with increases in the common structural glycans, Hex5HexNAc4 (shown in green) and Hex5HexNAc4dHex1 (shown in red). These increases correlated more with pathological changes than time post-exposure as can be observed by the increases in Hex5HexNAc4 (shown in green) in regions of significant pulmonary edema in the latent sample 040205 (middle left panel) and the end of study sample R03007 (bottom right panel). Similarly, Hex5HexNAc4dHex1 (shown in red), which was detected with increased intensity in regions of fibroconnective tissues surrounding the vessels and bronchi in control tissue, was detected with increased intensity in regions of interstitial fibrosis. These similarities and differences held true for all mucus-associated and pathology-specific glycan alterations detected. These results highlight the known complex nature of RILI and the molecular and cellular differences that occur within a sample and across samples following injury.

In addition to these alterations, two N-glycans were detected with decreased intensity in the lung parenchyma of IR samples at both time points analyzed. The high mannose glycan Hex5HexNAc2 and the hybrid/complex glycan Hex5HexNAc5dHex2 were both decreased following insult irrespective of time post-exposure. A further five N-glycans were decreased in only the end of study samples taken at 180 days post-exposure. Hex5HexNAc5dHex2, Hex6HexNAc6dHex1, Hex5HexNAc6dHex1, Hex2HexNAc7NeuAc2, and Hex6HexNAc5NeuAc1 were all decreased in the end of study samples but not the samples taken during the clinically latent period, indicating time-dependent alterations in the parenchyma (SF14).

## Discussion

The present study has identified alterations in a number of N-glycans that correlated with histological differences in pulmonary architecture following radiation insult and many of these alterations were independent of time post-exposure. Rather, the changes in N-glycan profiles appears to correlate with the pathological presentation of the pulmonary microenvironment. For example, the same increases in glycan compositions were observed in the early/premature fibrotic regions of 040205 and the more advanced and compact fibrotic regions of 061877, 04923, R03007 and 040087 for both the clinically latent period and the end of study time-points. Similar observations were observed for N-glycans that increased in regions of alveolar edema in the latent samples, 040205 and 061877 and the end of study sample, R03007, which had severe edema throughout the parenchyma. In these samples the increase observed is attributed to the presence of serum glycoproteins in the edema fluid.

Many samples had accumulations of alveolar macrophages that correlated with an increase in specific glycans, as shown in Figs. 2, 4 and 5. There has been renewed interest in pulmonary macrophages in recent years, particularly alveolar macrophages, and the role they play in pulmonary disease development. Specific focus has been on macrophage phenotype as these cells demonstrate extraordinary plasticity that is dependent on their surrounding microenvironment. A given phenotype can drive or resolve inflammation and fibrotic responses. Originally simplified into an M1/M2 paradigm that divides macrophages into classically and alternatively activated, in which the M1 phenotype is inflammatory and the M2 wound healing, and thus profibrogenic. It is now clear that the macrophage phenotype is more complex than just these dichotomous subtypes. A number of recent studies have placed alveolar macrophages as central to the development of pulmonary fibrosis in human idiopathic pulmonary fibrosis (IPF) cases, bleomycin models and following radiation injury^44–47^. The accumulation of alveolar macrophages was reported in most animals assayed in this and other models recently^7^. Their presence and involvement in the fibrotic and aberrant wound healing process that develops following radiation insult has been documented. The data presented here may also inform on the recent narrative in radiobiology that hypothesizes inflammation and fibrosis may be two separate, uninvolved entities. It has been shown that the standard corticosteroid treatment for inflammatory lung issues has little effect on macrophage numbers in the lung and some studies have shown it may actually increase the shift to the M2 phenotype and thus exacerbate the profibrotic process. Furthermore, macrophages collected from patients with chronic obstructive pulmonary disease (COPD) were shown to be insensitive to corticosteroid-induced immunosuppression since release of inflammatory cytokines from these macrophages was not mitigated by exposure to dexamethasone^48^. There is repeated demonstration that tissue-resident alveolar macrophages (TR-AM) are depleted following radiation injury and replaced with monocyte-derived alveolar macrophages (MO-AM)^46,47^. MO-AMs were shown to be the major cells responsible for the profibrotic response in bleomycin-induced fibrosis in mice and in human patients with end-stage fibrotic diseases that include IPF, systemic sclerosis-associated interstitial lung disease (SSc-ILD), mixed connective tissue disease (MCTD), and hypersensitivity pneumonitis (HP)^44^. The study by Misharin et al., also demonstrated amelioration of fibrosis following depletion of MO-AMs but not TR-AMs^44^. The authors also provided evidence to show that dexamethasone had little-to-no impact on both TR-AMs and MO-AMs. Their findings provide possible insight into the questions of why dexamethasone has little utility toward the resolution of RILI and has adverse effects when used to treat patients with IPF.

In addition to the pathological changes and the accumulations of N-glycans to distinct cellular populations or regions of edema, the accumulation of mucus within alveolar and bronchiolar space has enabled the assignment of N-glycans to secreted mucus. The assignment of mucus-associated N-glycans would not have been possible in control tissue alone due to the number of cells and the molecular complexity of the parenchyma. The normal pulmonary architecture is complex with many cell types, mucus secretions and surfactant, thus assigning N-glycans to these individual components would not have been possible without significant localized increases due to pathological alterations. The mucus accumulation as a result of alveolar and alveolar-bronchiolar hyperplasia that was observed in sample 04923 enabled the identification of mucus-associated N-glycans. Much of the glycosylation data for pulmonary mucus focuses on O-linked glycans from mucin proteins and there is very little on the role of N-linked glycans in pulmonary mucus to date, this warrants further investigation^49^.

In summary, this study has identified the localization of a number of N-glycans in the normal pulmonary parenchyma and following the multiple complex alterations that develop following RILI. Future studies could include interrogation of the functional roles these glycans play, with a particular focus on macrophage accumulation and phenotype, as this may be an important therapeutic avenue for the mitigation of pulmonary fibrosis following RILI.

## Materials and method

### Materials

Analytical grade solvents, acetonitrile (ACN) and water (H_2_O) were purchased from Fisher Scientific (Pittsburgh, PA, USA). Histological grade xylene and ethanol, and the MALDI matrix, alpha-cyano-4-hydroxycinnamic acid (CHCA) were purchased from Sigma Aldrich (St. Louis, MO). Hematoxylin and eosin (H&E) staining kit, citraconic anhydride for antigen retrieval and positively charged glass slides were purchased from Thermo Scientific (San Jose, CA, USA). Recombinant Peptide N-Glycosidase F (PNGase F) was obtained from the laboratory of Dr. Richard Drake (Charleston, NC, USA) and the commercial version, PNGase F PRIME™, was purchased from N-zyme Scientifics (Doylestown, PA, USA).

### Methods

#### Partial-body irradiation model

All animal procedures were conducted in accordance with the NIH guidelines for the care and use of laboratory animals and experiments were performed with prior approval from the University of Maryland Institutional Animal Care and Use Committee (IACUC). Details of the animal model, irradiation and medical management for the partial-body irradiation model with 5% bone marrow sparing (PBI/BM5) have been described in detail previously^37^. Briefly, male Chinese rhesus macaques (Macaca mulatta, 5.3–10.4 kg) were exposed to 10.0 Gy utilizing 6 MV LINAC-derived photons at a dose rate of approximately 0.80 Gy min^−1^ (TrueBeam™, Varian Medical Systems, Palo Alto, CA). Dose was delivered to midline tissue, half of the prescribed dose was delivered with an anteroposterior (AP) beam, and half with a posteroanterior (PA) beam. Dosimetry was calculated using standard clinical protocols based on baseline CT scans of the tissue area to include a 2 cm margin beyond the lungs. Dosimetry calculations and a retrospective dose-reconstruction study have been described in detail previously^50,51^. Medical management was provided on an individual basis based on a set of clinical triggers and included, hydration, antibiotics, analgesics, anti-diarrheals, antipyretics, anti-emetics, anti-ulceratives, nutritional support, and blood transfusions. Full details of the administration of medical management and treatment duration has been previously published^37^. Animals in respiratory distress, defined as a nonsedated respiratory rate greater than 80 breaths per minute, were treated with a planned taper of dexamethasone as previously published^3^.

Animals were euthanized at the planned end of study, 180 (± 5) days or earlier if the animal met the IACUC-approved alternate endpoint criteria. Lung samples collected during the clinically latent period (~ 50 ± 10 days) of the pulmonary syndrome were from animals that had succumbed to the acute radiation syndrome of the hematopoietic system (H-ARS) plus gastrointestinal (GI) and kidney damage^38,39^. The samples taken at the end of study (EoS) were from animals that survived GI-ARS and H-ARS, prolonged GI damage, kidney damage, pulmonary syndrome and multi-organ injury.

#### Tissue preparation and PNGase F application

Study design was such that control and IR slides were prepared together as paired samples and acquired simultaneously, alternating the order of acquisition across samples and for technical replicates. Tissue preparation and PNGase F application was performed according to the recently published protocol^52^. FFPE lung blocks were sectioned at 3 µm thickness using a Shandon Finesse microtome (Thermo Scientific, Waltham, MA). Slides were heated at 60 °C for 1 h. Slides were dewaxed and rehydrated for heat-induced antigen retrieval. One slide each from control and IR samples were placed in a 5-slide mailer with a solution of citraconic anhydride buffer and antigen retrieval was carried out for 30 min using a Hamilton Beach vegetable steamer. For PNGase F application, 100 µg of enzyme was deposited over slides using the TM-Sprayer™ (HTX Technologies, Chapel Hill, NC). Deposition was 30 µL/minute, 15 passes, crisscross pattern, velocity of 1200, and 3.0 mm track spacing, and the nitrogen gas was set to 11 psi. Slides were then placed in a preheated incubation chamber and allowed to incubate for 2.5 h at 37 °C.

#### Matrix deposition and MSI acquisition

CHCA at a concentration of 5 mg/mL in 50% ACN was deposited over the tissue sections using the TM-Sprayer™ with the following settings: 100 µL/min, 10 passes, crisscross pattern, velocity of 1300, 3 mm track spacing and 11 psi. Imaging experiments were performed using a 12 T Solarix XR Fourier-transform ion cyclotron resonance (FTICR) mass spectrometer (Bruker Daltonics, Bremen, Germany) equipped with a dual ESI/MALDI ion source and a Smartbeam II Nd:YAG (355 nm) laser. The instrument was operated in positive ion mode over an *m/z* range of 498–5000 with an estimated resolving power of 130,000 at *m/z* 400. The sample plate stepping distance was set to 50 μm using the random walk setting. The laser power was set to 17% with 200 shots per pixel using the small laser setting (~ 50 μm diameter). The ion at *m/z* 1809.6396 (Hex5dHex1HexNAc4) was used for lock mass as this N-glycan is commonly detected across all tissue regions.

#### Data analysis

Data analysis was carried out in FlexImaging 4.0 and the SCiLs Lab software suite version 2016b (Bruker Daltonics). Data files from control and IR samples were combined and imported as a single file into the SCiLS Lab software for comparison. Intensity box plots were generated to show the distribution of intensity within the different pathological regions detected. All data presented in the intensity box plots had a receiver operating characteristic area under the curve ROC AUC of at least 8.5. N-glycan images were generated and overlaid with their histological sections for anatomy and pathology-specific distribution using the FlexImaging software. Region and disease-specific single pixel spectra were exported from FlexImaging and opened in the Data Analysis software version 4.4 (Bruker Daltonics) for analysis. The average ion spectrum for each data set was imported into GlycoWorkBench for N-glycan analysis^53^. Identification of N-glycans were based on the structures identified in GlycoWorkBench and those previously published^24,31,52^.

#### Histological staining

Following image acquisition, the sections were washed with 70% EtOH for 1 min and 100% EtOH for 1 min. Slides were stained with hematoxylin and eosin (H&E) using the manufacturers’ protocol. Slides were then scanned using the Aperio slide scanner (Leica Microsystems Inc., IL, USA) and images were imported into their corresponding FlexImaging datasets for correlation with the MALDI-MS data for N-Glycan distribution.

## Supplementary information

Supplementary information.
