# Supplementary material for: MALDI-MSI spatially maps N-glycan alterations to histologically distinct pulmonary pathologies following irradiation
Source: Sci Rep. 2020 Jul 14;10:11559. doi: 10.1038/s41598-020-68508-y (PMC7360629; doi:10.1038/s41598-020-68508-y)
Supplement: Supplementary file 1 — Supplementary information. [file 41598_2020_68508_MOESM1_ESM.pdf]

MALDI-MSI spatially maps N-glycan alterations to histologically distinct pulmonary pathologies following irradiation

Claire L. Carter[1], George Parker[3], Kim G. Hankey[2], Ann M. Farese[2], Thomas J. MacVittie[2], and Maureen A. Kane[1]*

1) University of Maryland, School of Pharmacy, Department of Pharmaceutical Sciences, Maryland, USA
2) University of Maryland, School of Medicine, Department of Radiation Oncology, Maryland, USA
3) Charles River Laboratories, Pathology Associates, Raleigh-Durham, North Carolina, USA

***Correspondence:**

Maureen A. Kane, Ph.D

Department of Pharmaceutical Sciences

University of Maryland School of Pharmacy

20 N. Pine Street, Room N706

Baltimore, MD 21201

Phone: 410-706-5097

Fax: 410-706-5017

Email: mkane@rx.umaryland.edu

SUPPLEMENTARY MATERIAL

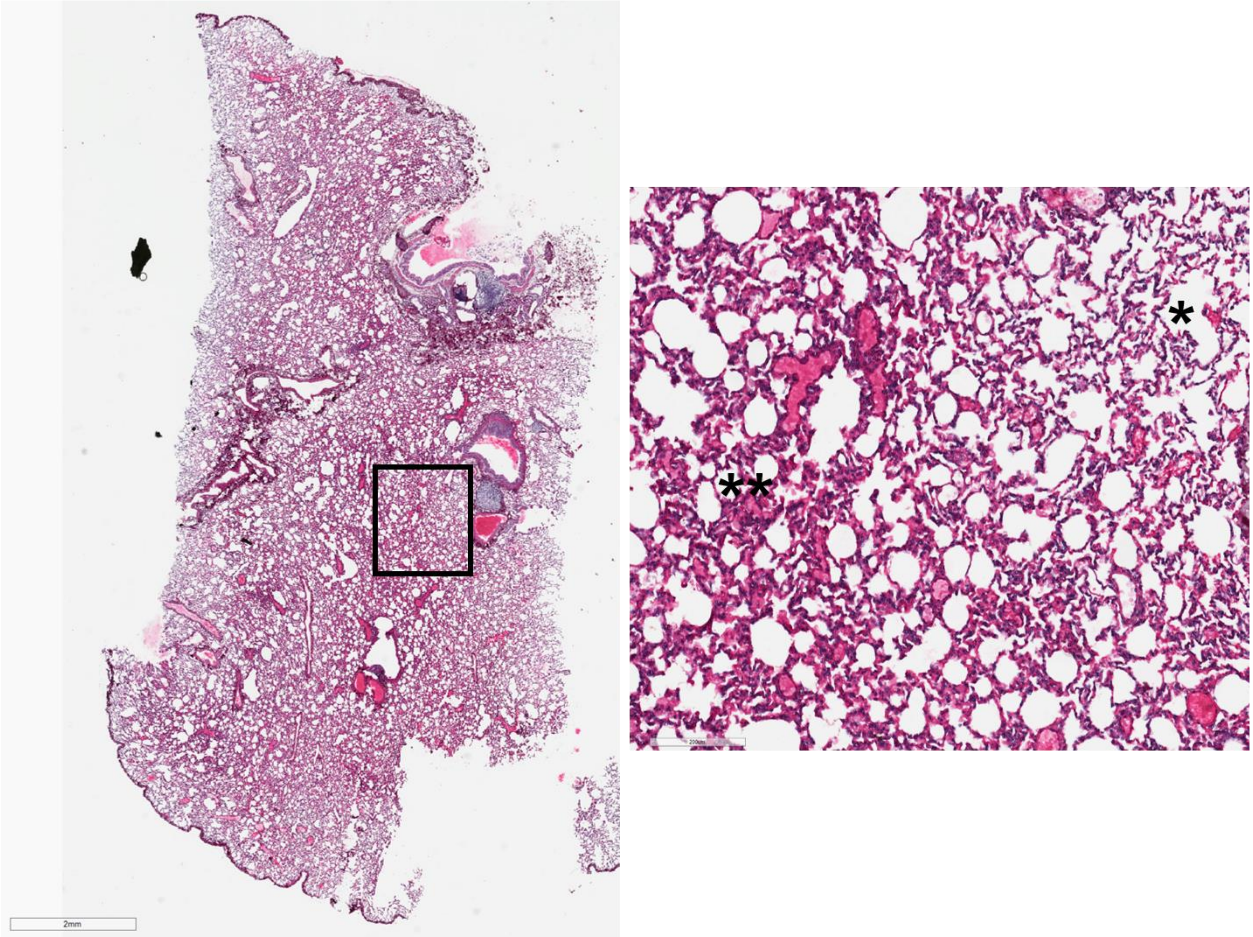

Figure S1. Histology at 50 days post-exposure. H&E stained section of control sample 0879. The image on the right is taken from the region highlighted by the square box and shows regions of collapsed lung (**) and regions of inflated lung architecture (*).

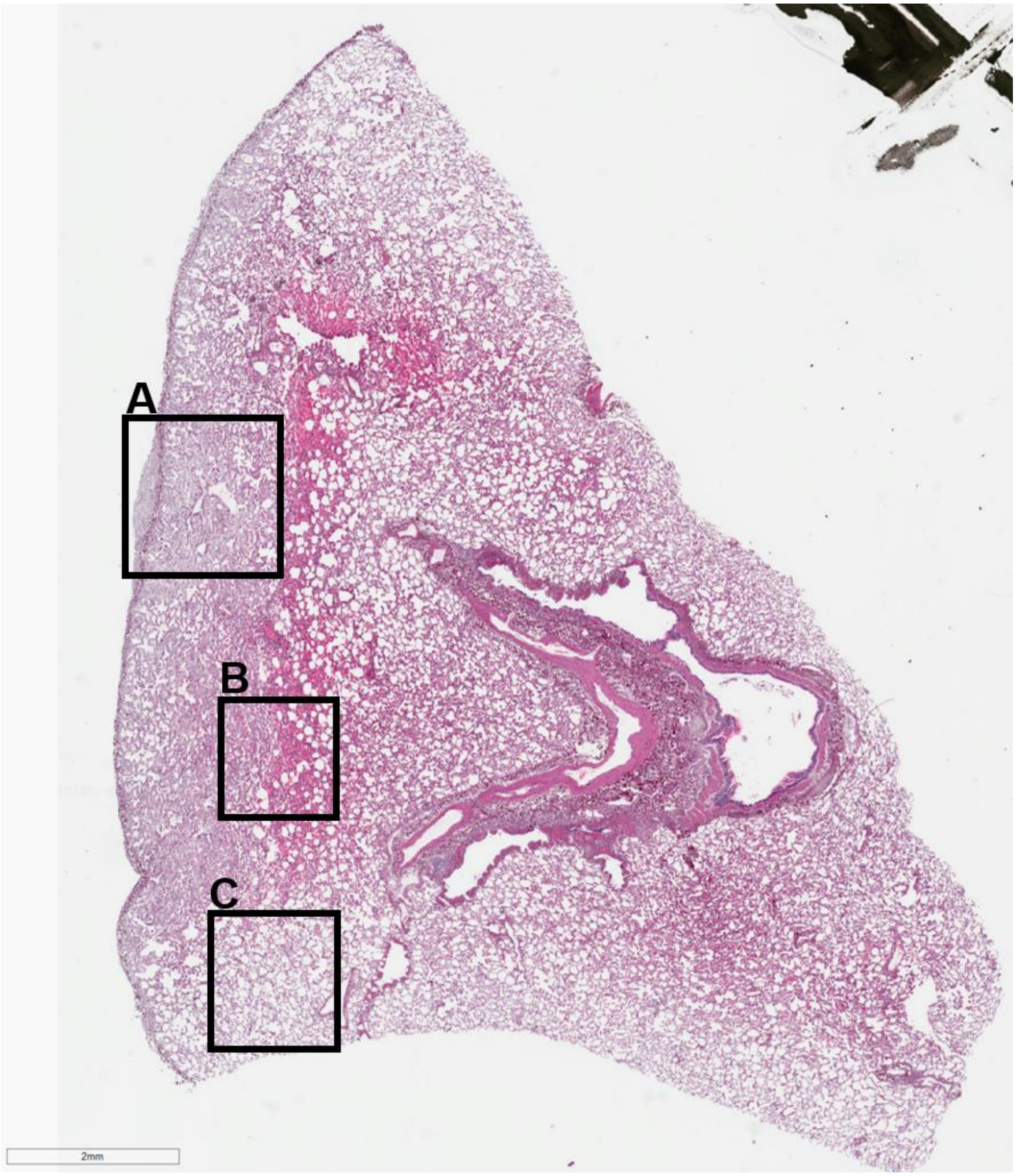

Figure S2. Histology at 50 days post-exposure. H&E stained section of irradiated animal, 040205. The boxes are representative regions of the differing pathologies observed in this animal at 50 +/- 10 days post-irradiation and are described below.

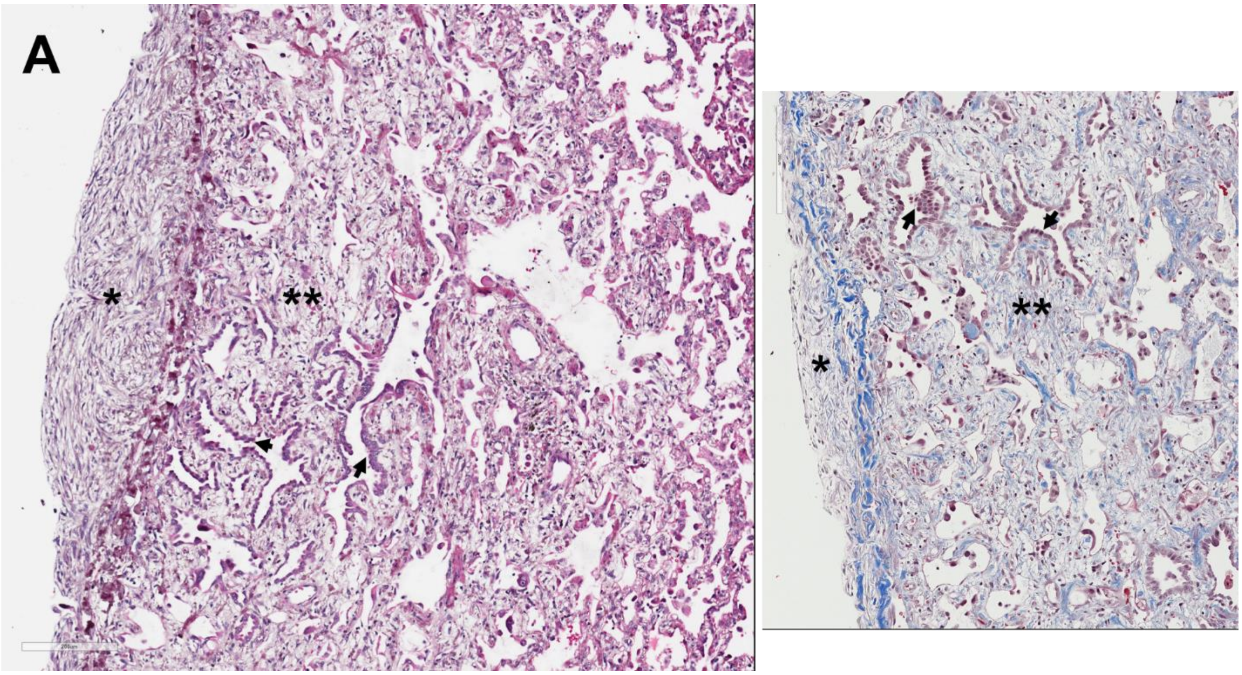

Figure S2A. Histology at 50 days post-exposure. Higher magnification image of region A taken from 040205. The original pleural layer of dense fibrous connective tissue is covered by a thick layer of immature fibrous connect tissue (*). Subpleural alveoli also shows evidence of fibrosis with immature fibrous connective tissue (**). This is further evidenced by the trichrome section on the right, taken from the same sample but several hundred microns away from that used in the MSI study. Immature fibrous connective tissue is demonstrated by light blue staining. Hyperplasia of alveolar and/or bronchiolar epithelial cells is shown (arrows).

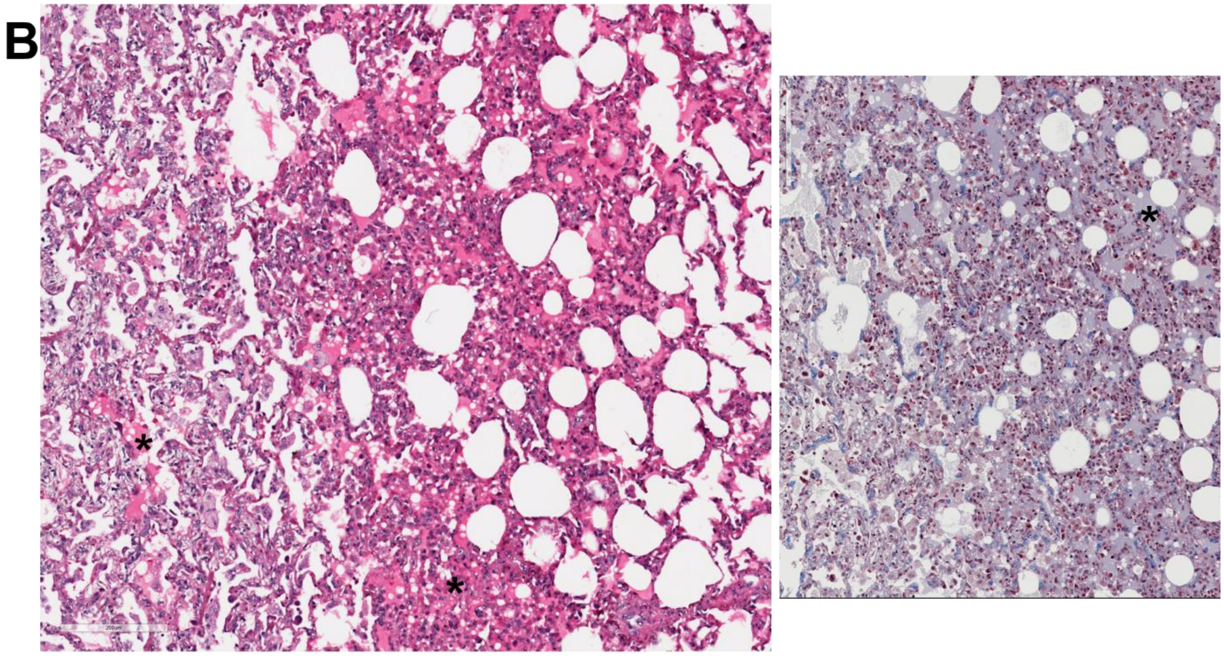

Figure S2B. Histology at 50 days post-exposure. Higher magnification image of region B taken from 040205. Note the presence of edema (*) that ran parallel to the immature fibrosis region.

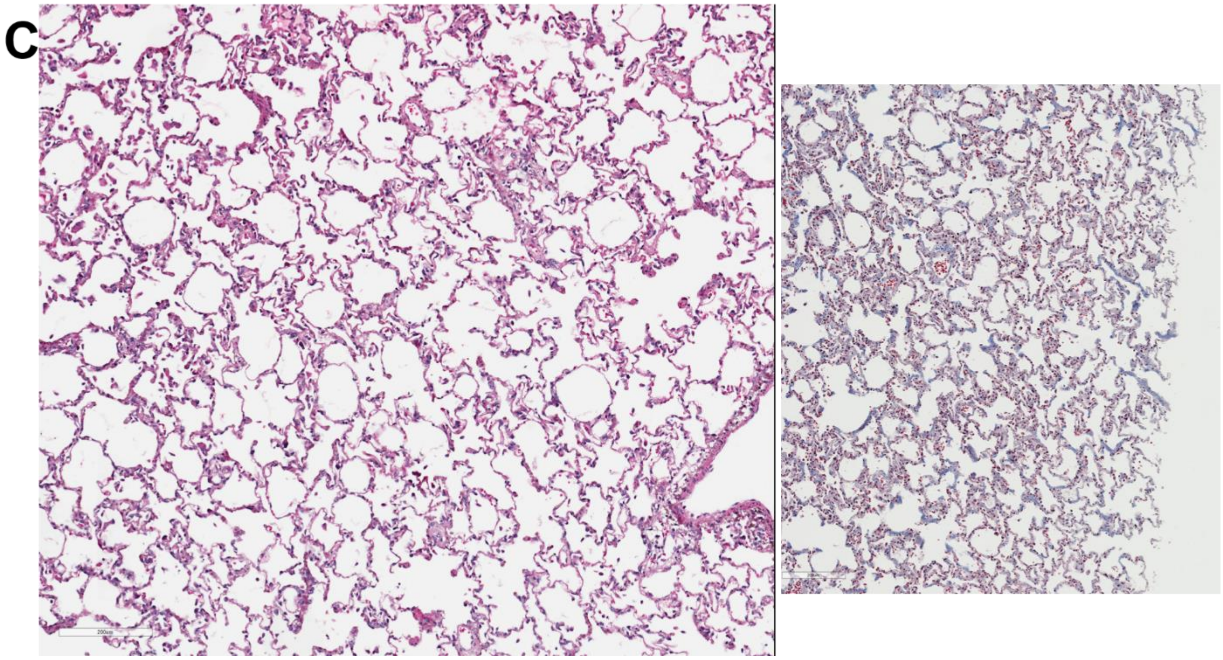

Figure S2C. Histology at 50 days post-exposure. Higher magnification image of region C taken from 040205. Note the normal appearing pulmonary architecture.

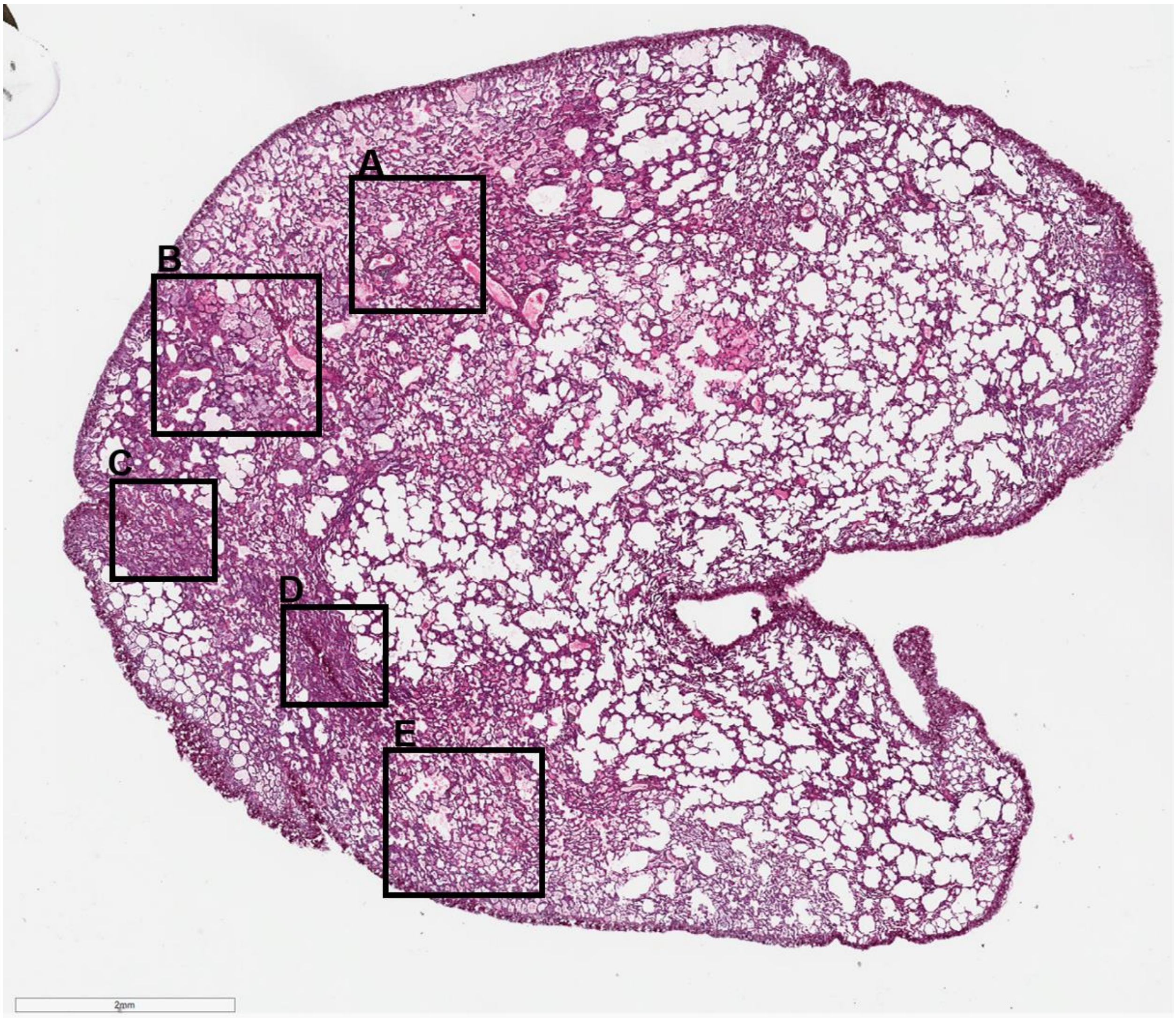

Figure S3. Histology at 50 days post-exposure. H&E stained section of irradiated animal, 061877. The boxes are representative regions of the differing pathologies observed in this animal at 50 +/- 10 days post-irradiation and are described below.

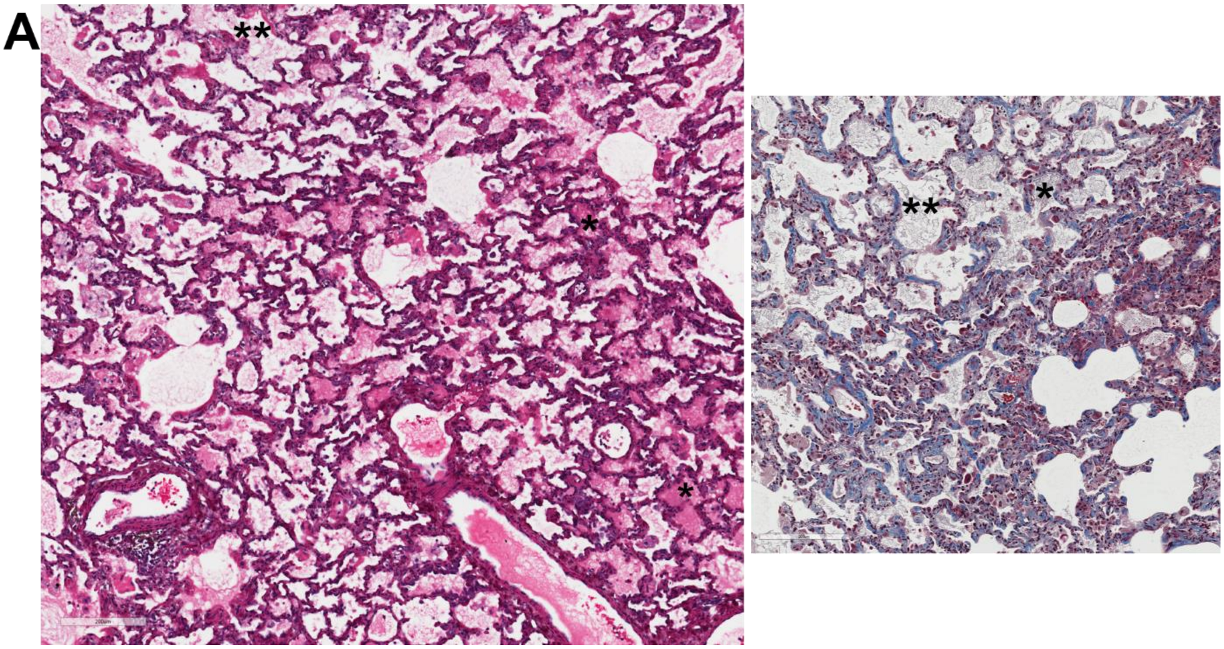

Figure S3A. Histology at 50 days post-exposure. Higher magnification image of region A taken from 061877. Note the presence of edema (*) and mucinous material (**) in the alveolar space. The trichrome section on the right is again from the same sample but several hundred microns away. Interstitial fibrosis is evident on both sections but highlighted readily by the blue staining of collagen deposition in the right section.

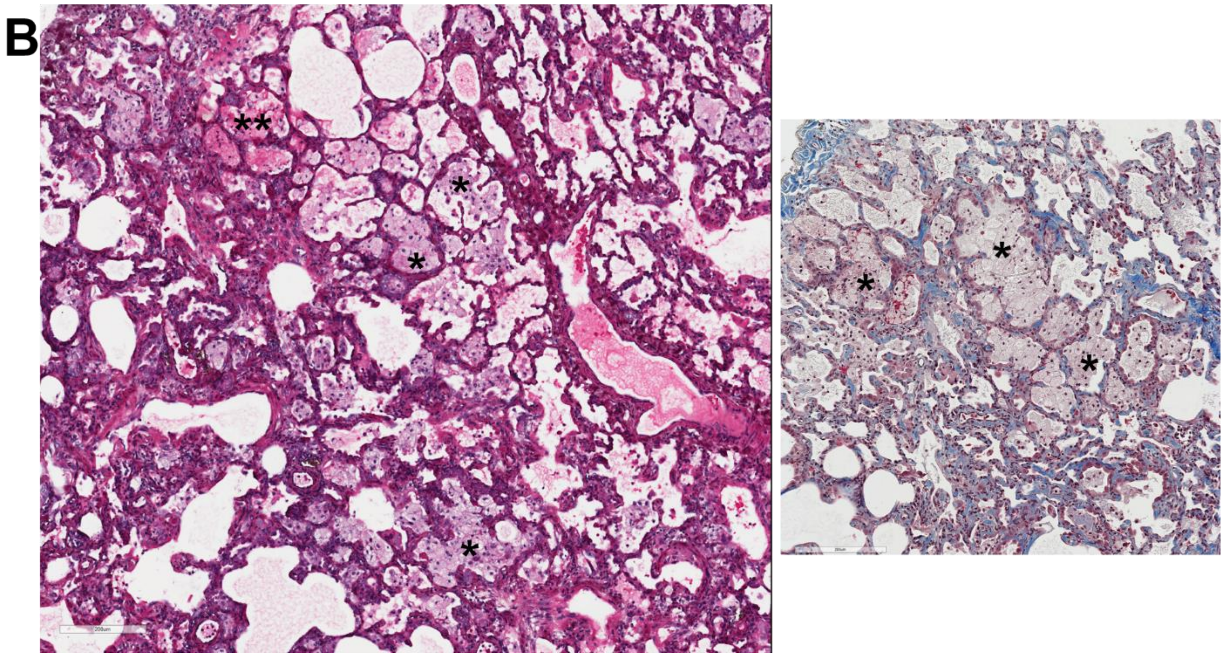

Figure S3B. Histology at 50 days post-exposure. Higher magnification image of region B taken from 061877. Note the presence of alveolar macrophage accumulation (*) and edema (**) in the alveolar space.

C

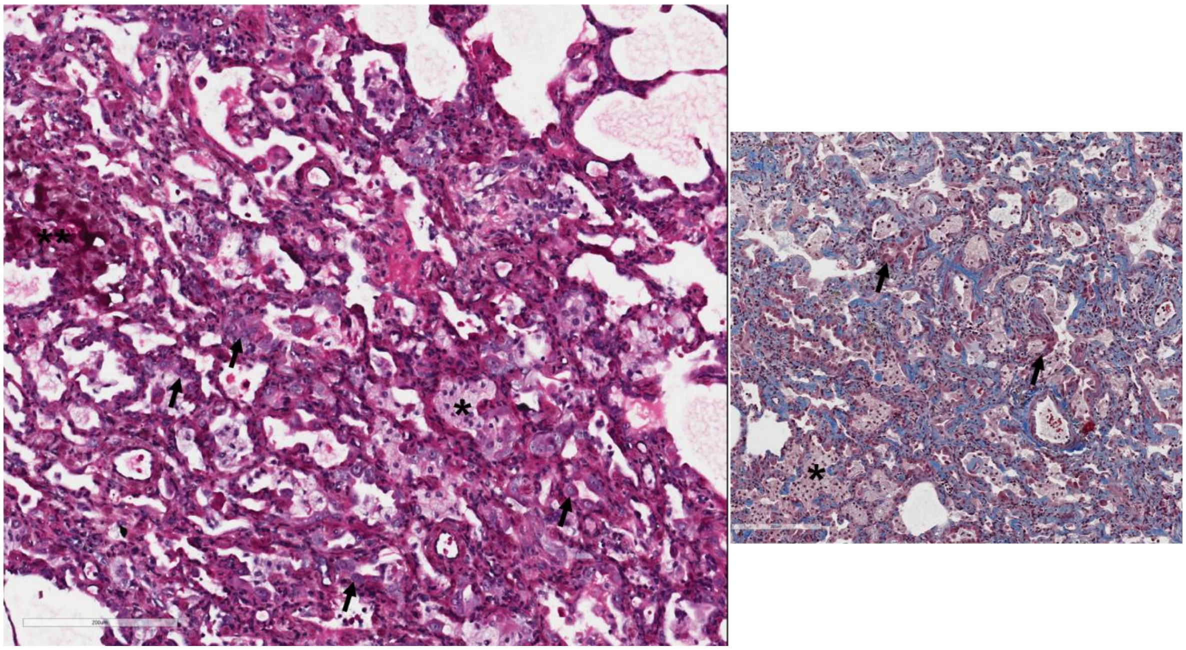

Figure S3C. Histology at 50 days post-exposure. Higher magnification image of region C taken from 061877. Note the presence of alveolar macrophage accumulation (*). Hyperplasia of type 2 alveolar epithelial cells and alveolar-bronchiolar hyperplasia is evident; examples are shown (arrows). Thickening of alveolar walls is evident and further demonstrated by the blue collagen staining in the trichrome section on the right. The trichrome section is from the same sample but several hundred microns from that used in the MSI studies and the H&E presented on the left.

D

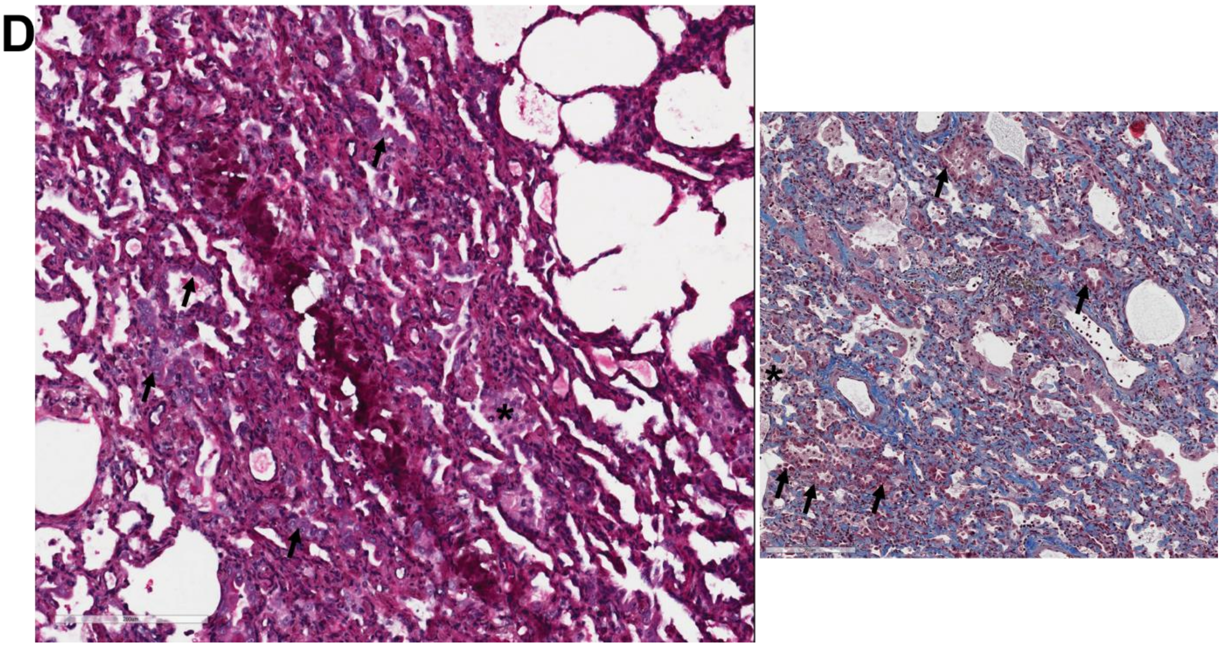

Figure S3D. Histology at 50 days post-exposure. Higher magnification image of region D taken from 061877. Note the presence of hyperplasia of type 2 alveolar epithelial cells and alveolar-bronchiolar hyperplasia (arrows).

E

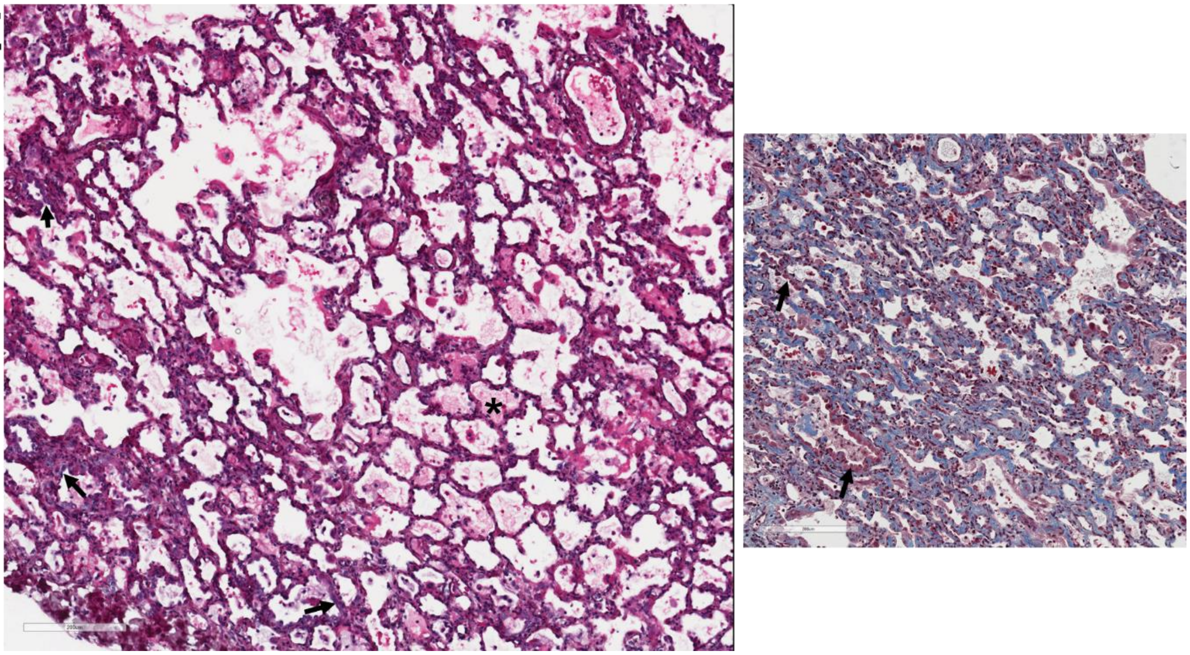

Figure S3E. Histology at 50 days post-exposure. Higher magnification image of region D taken from 061877. Note the presence of edema (*), hyperplasia of type 2 alveolar epithelial cells, and alveolar-bronchiolar hyperplasia (arrows).

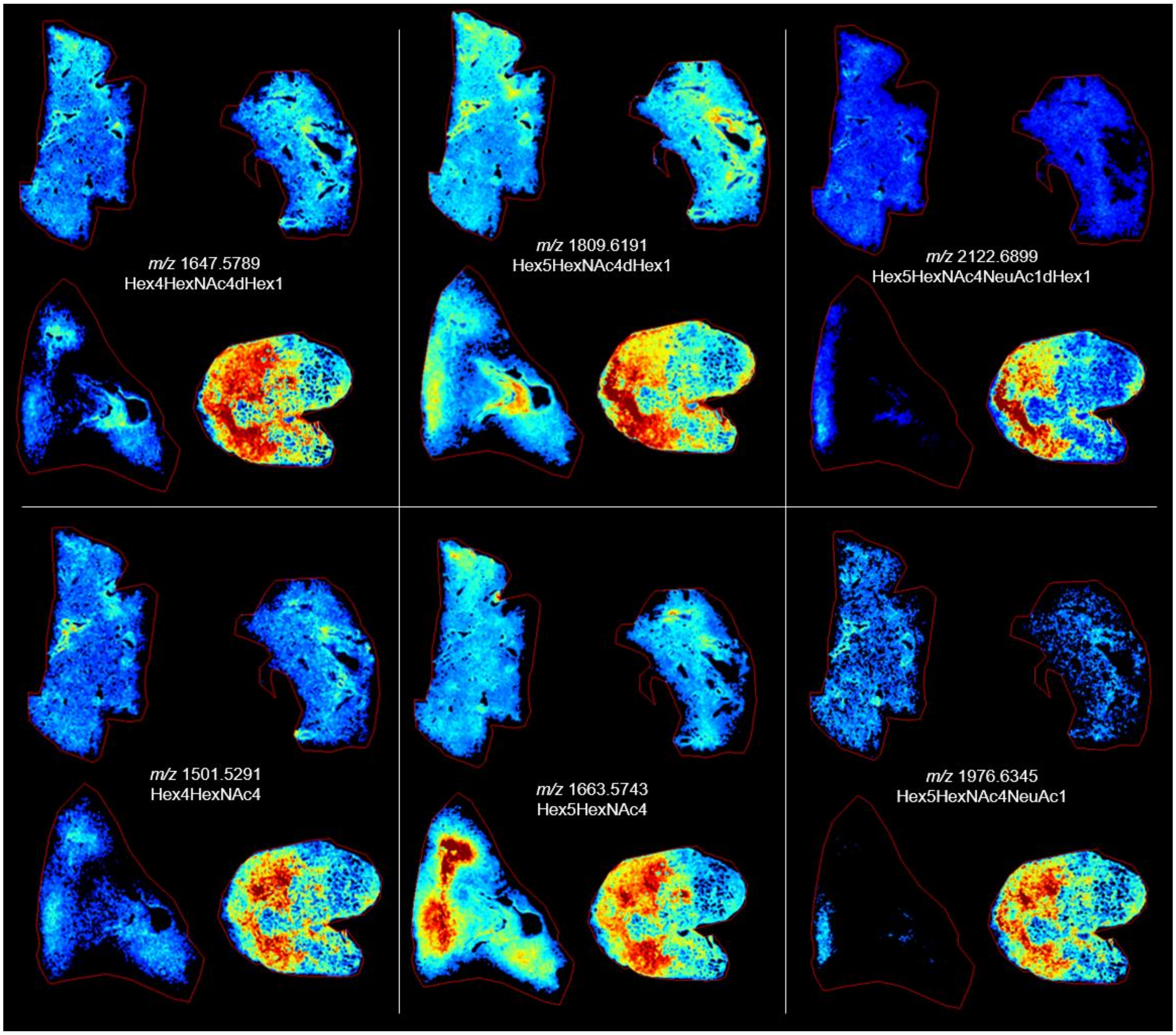

Figure S4. MSI at 50 days post-exposure. MALDI MS images of N-glycans that were detected with regional increases in intensity during the clinically latent period. The top panels are core fucosylated N-glycans.

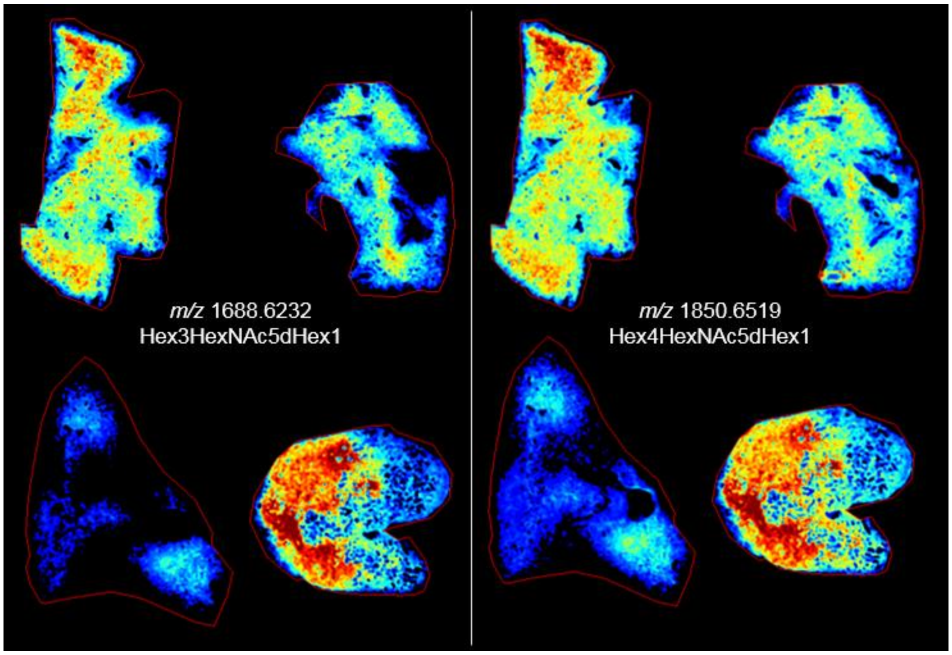

Figure S5. MSI at 50 days post-exposure MALDI MS images of N-glycans that were detected with regional increases in intensity during the clinically latent period. The top panels are core fucosylated N-glycans.

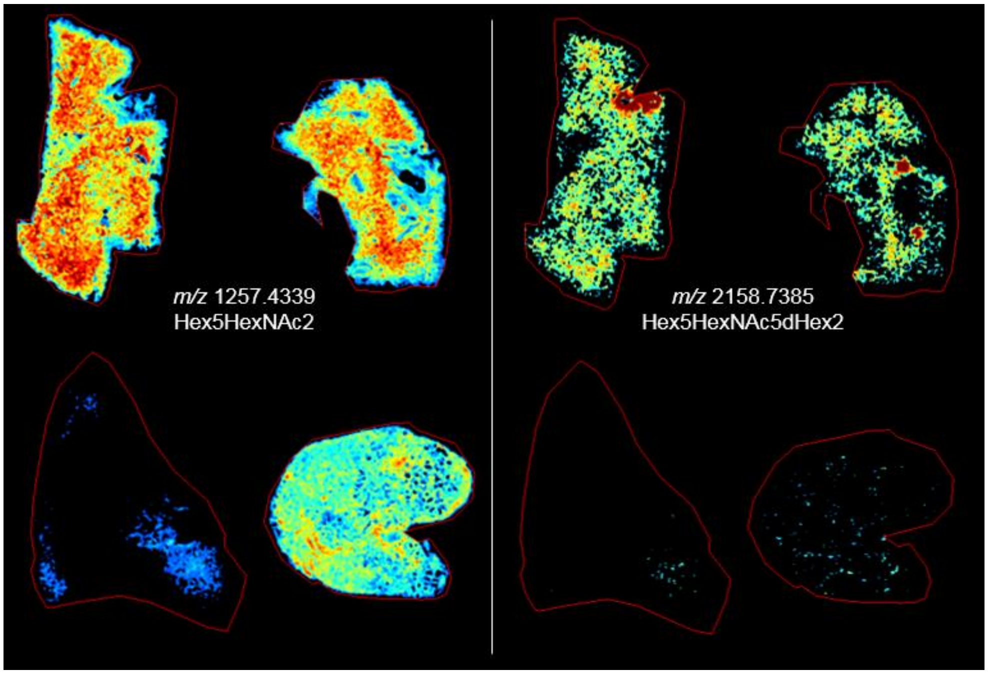

Figure S6. MSI at 50 days post-exposure MALDI MS images of N-glycans that were detected with decreased intensity during the clinically latent period.

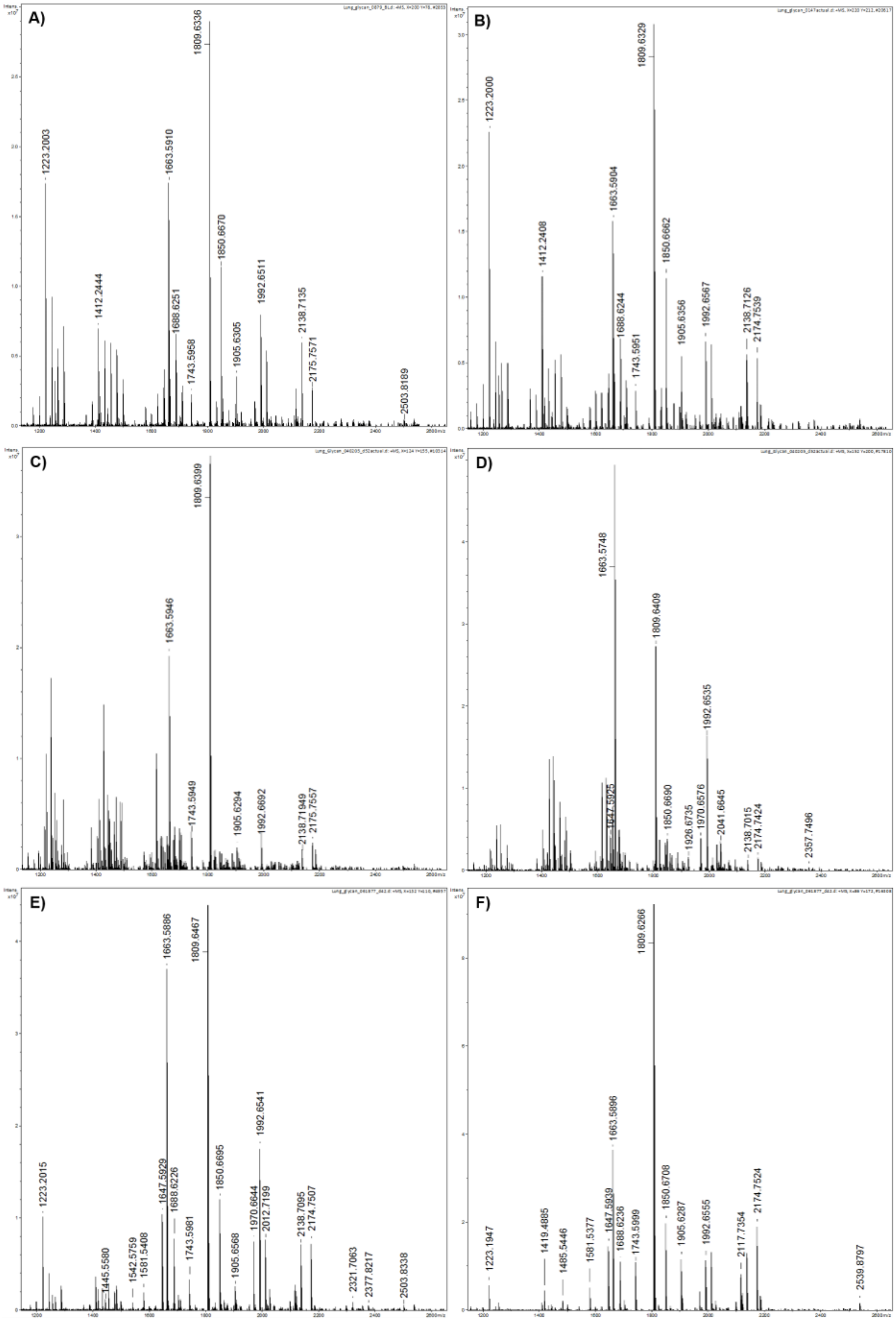

Figure S7. Mass spectra at 50 days post-exposure. Representative MALDI-MS spectra taken from the control sections 0879 (A) and 0147 (B), IR 040205 immature subpleural fibrosis (C) and edema (D), and IR 061877 edema (E) and regions of alveolar macrophage accumulation, AEC2 hyperplasia and fibrosis.

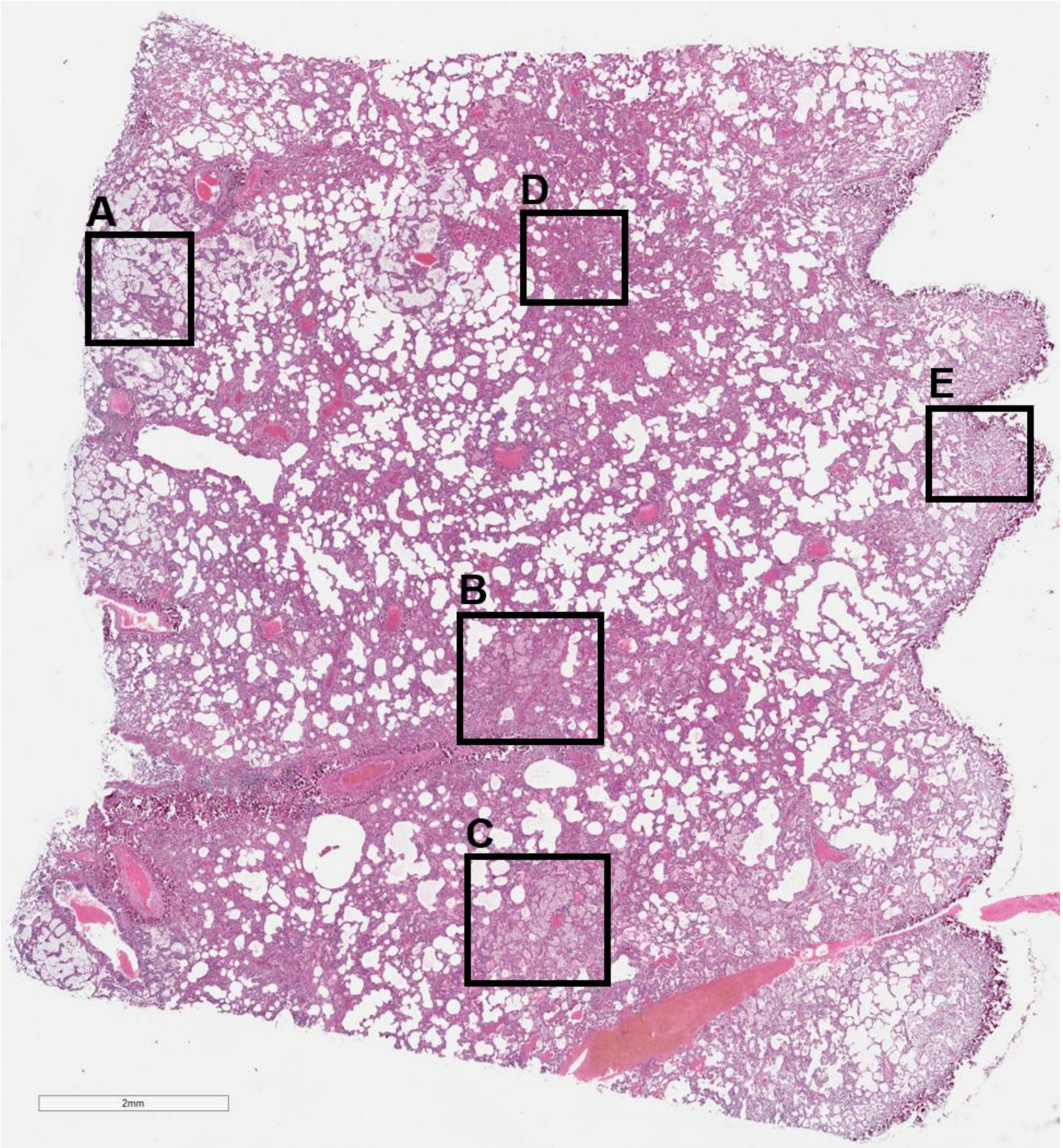

Figure S8. Histology at 180 days post-exposure. H&E stained section of irradiated animal, 04923. The boxes are representative regions of the complex pathologies observed in this animal at 180 days post-irradiation and are described below.

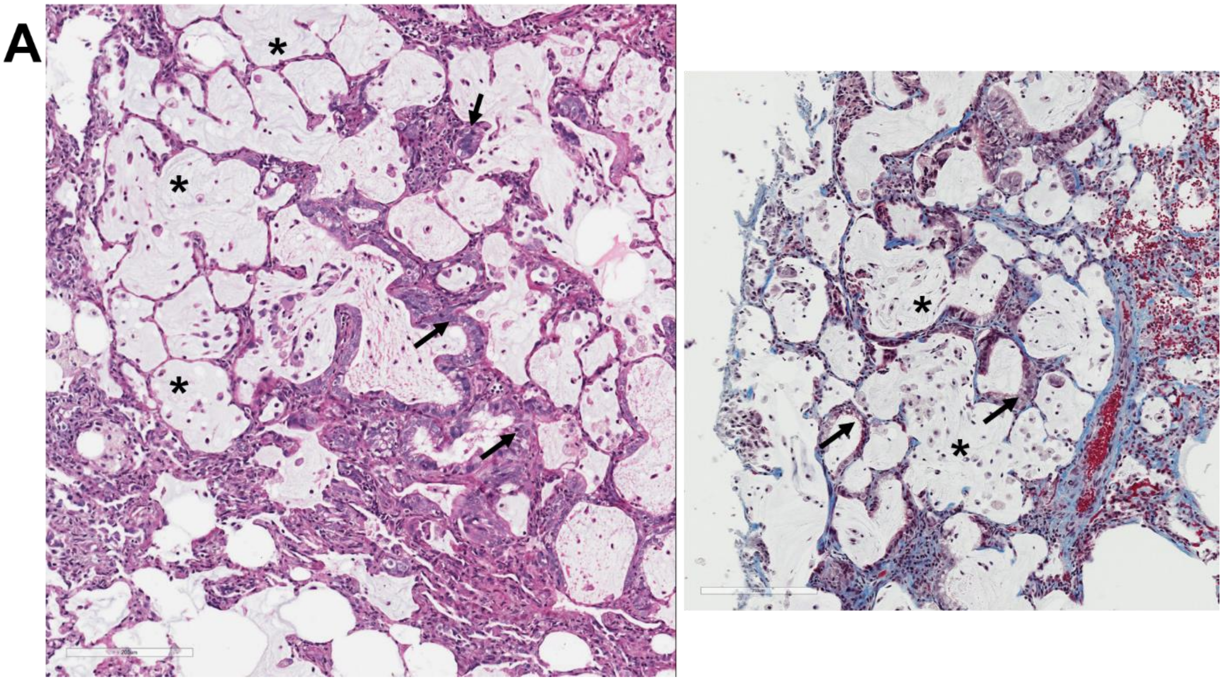

Figure S8A. Histology at 180 days post-exposure. Higher magnification image of region A taken from 04923. This region shows mucus accumulation within the alveolar and bronchiolar space (*) along with alveolar and alveolar-bronchiolar hyperplasia (arrows). The trichrome section on the right is from the same sample but taken several hundred microns from the sections used for MSI and its H&E section on the left.

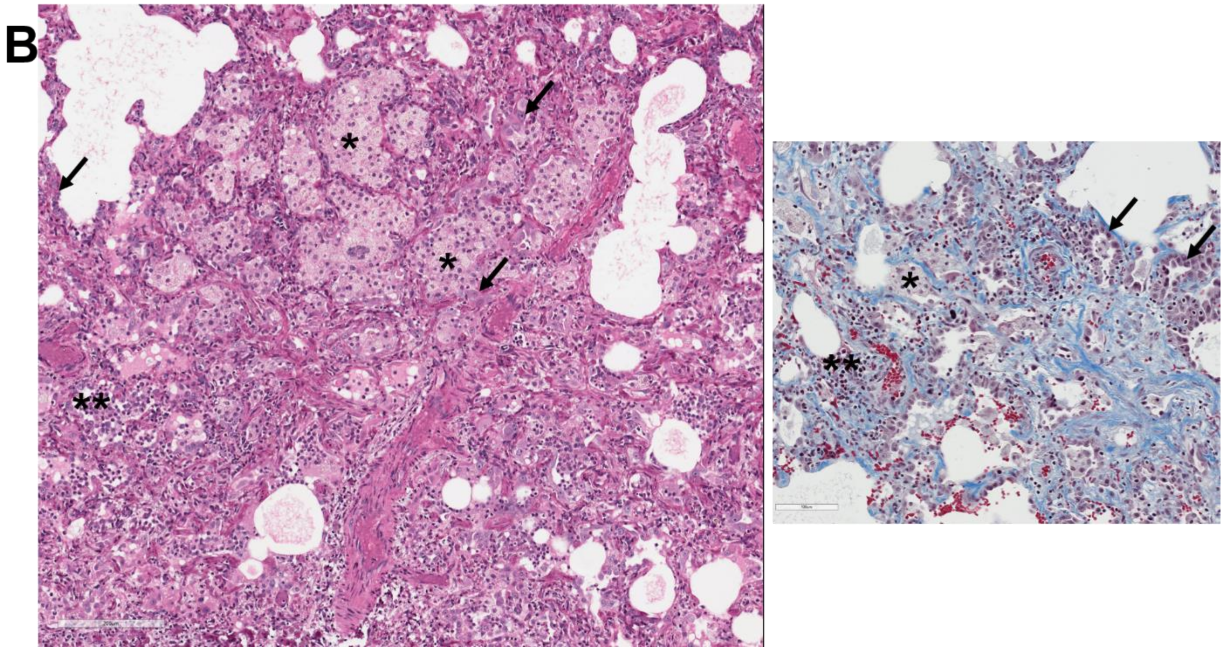

Figure S8B. Histology at 180 days post-exposure. Higher magnification image of region B taken from 04923. Note the accumulations of alveolar macrophages (*) and mixed immune infiltrations, predominantly lymphocytes (**) within the alveolar lumen. Alveolar and alveolar-bronchiolar hyperplasia (arrows). The thickened fibrotic alveolar walls are evident and further demonstrated by the blue collagen staining in the image on the right. This trichrome section is from the same sample but taken several hundred microns from the sections used for MSI and its H&E section on the left.

C

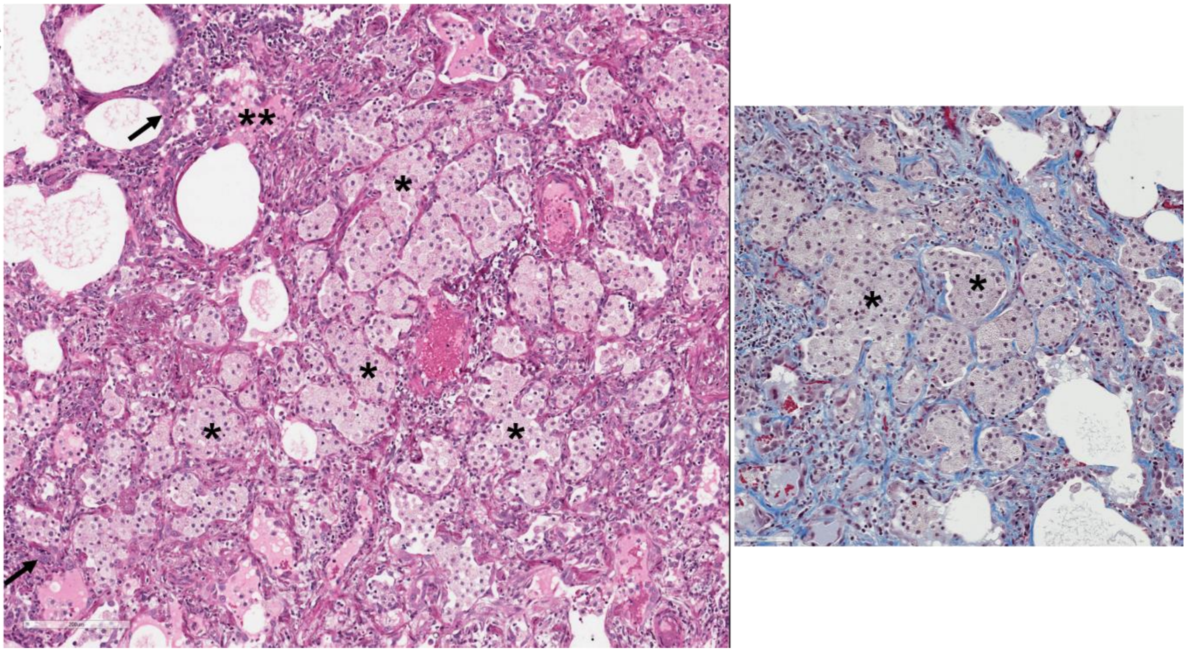

Figure S8C. Histology at 180 days post-exposure. Higher magnification image of region C taken from 04923. Note the accumulations of alveolar macrophages (*) within the alveolar lumen and alveolar and alveolar-bronchiolar hyperplasia (arrows). The thickened fibrotic alveolar walls are evident and further demonstrated by the blue collagen staining in the image on the right. This trichrome section is from the same sample but taken several hundred microns from the sections used for MSI and its H&E section on the left.

D

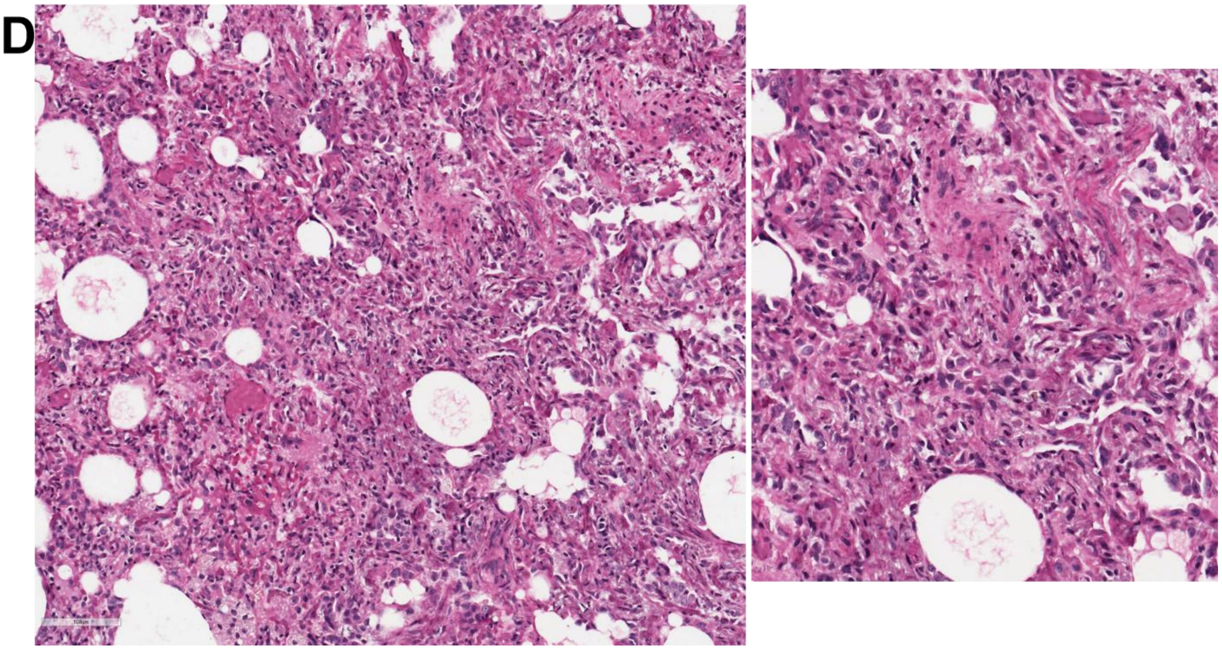

Figure S8D. Histology at 180 days post-exposure. Higher magnification image of region D taken from 04923.

E

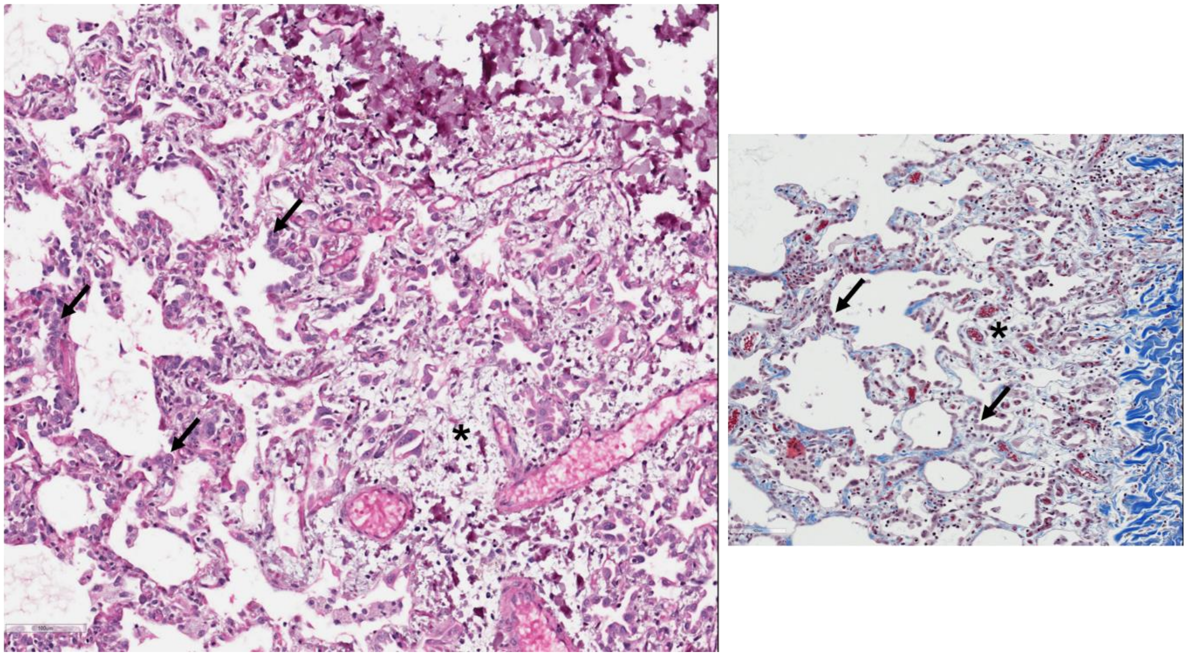

Figure S8E. Histology at 180 days post-exposure. Higher magnification image of region E taken from 04923. Subpleural alveoli structure is lost due to layers of immature fibrous connective tissue (*). Note alveolar and alveolar-bronchiolar hyperplasia (arrows). This trichrome section is from the same sample but taken several hundred microns from the sections used for MSI and its H&E section on the left.

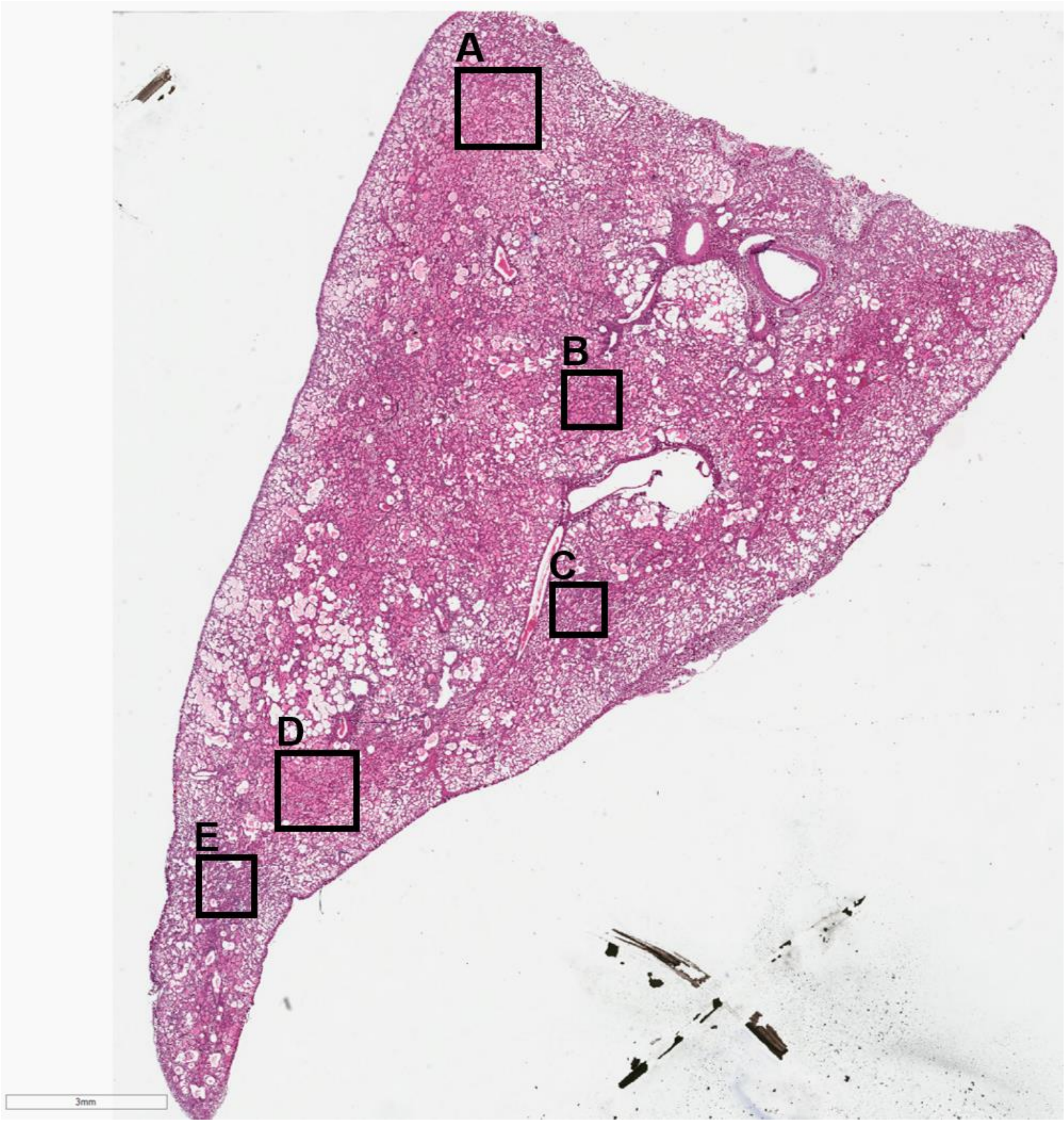

Figure S9. Histology at 180 days post-exposure. H&E stained section of irradiated animal, R03007. The boxes are representative regions of the complex pathologies observed in this animal at 180 days post-irradiation and are described below.

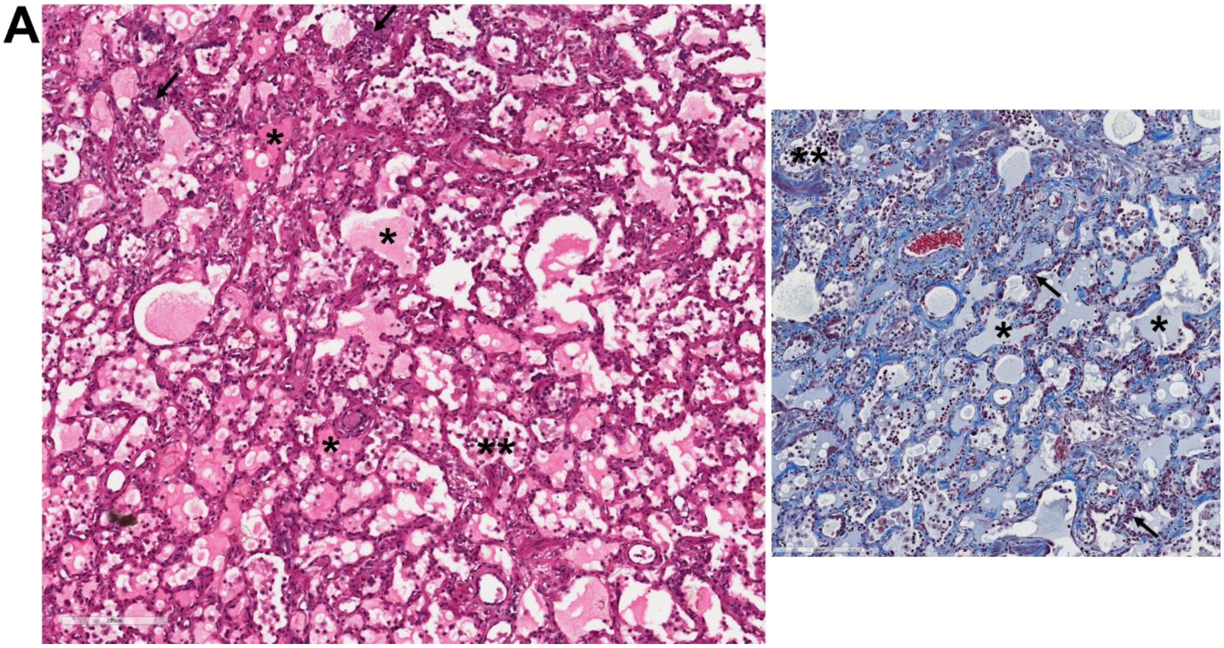

Figure S9A. Histology at 180 days post-exposure. Higher magnification image of region A taken from R03007. Note the presence of edema (*) and mixed immune infiltration, predominantly lymphocytes (**), in the alveolar space. The trichrome section on the right is again from the same sample but several hundred microns away. Interstitial fibrosis is evident on both sections but highlighted readily by the blue staining of collagen deposition in the right section.

B

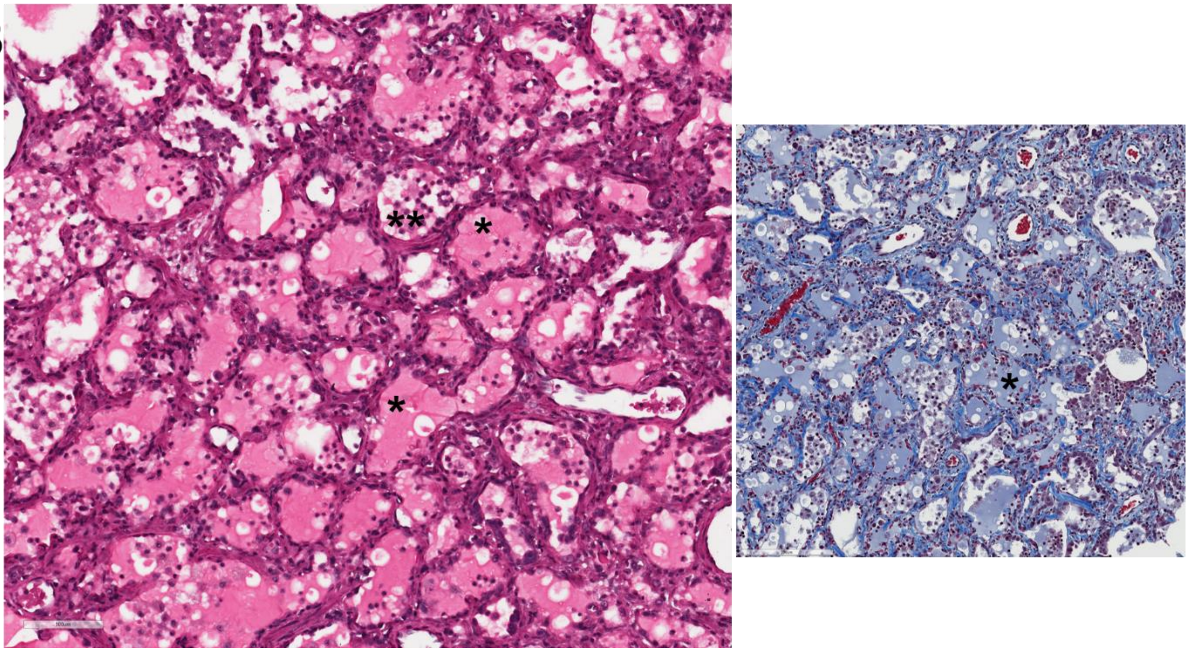

Figure S9B. Histology at 180 days post-exposure. Higher magnification image of region B taken from R03007. Note the presence of edema (*) and mixed immune infiltration, macrophages and lymphocytes (**), in the alveolar space. The trichrome section on the right is again from the same sample but several hundred microns away. Interstitial fibrosis is evident on both sections but highlighted readily by the blue staining of collagen deposition in the right section.

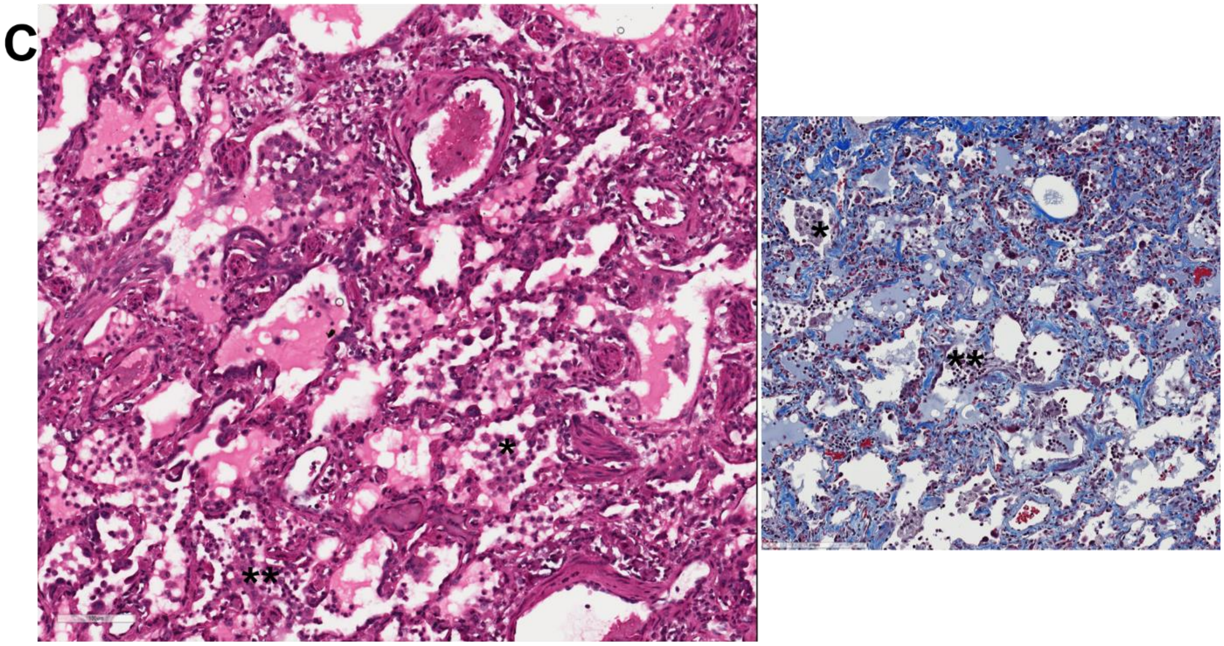

Figure S9C. Histology at 180 days post-exposure. Higher magnification image of region C taken from R03007. Note the presence of alveolar macrophage accumulation (*) and mixed immune infiltration, predominantly lymphocytes (**), in the alveolar space. The trichrome section on the right is again from the same sample but several hundred microns away. Interstitial fibrosis is evident on both sections but highlighted readily by the blue staining of collagen deposition in the right section.

D

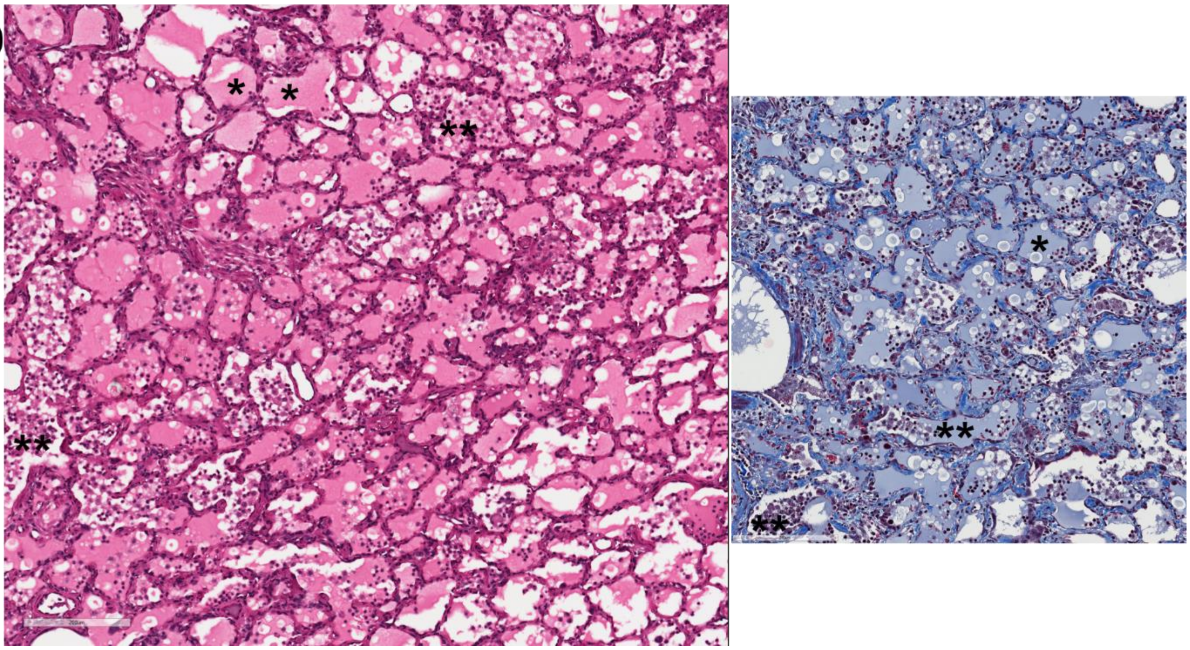

Figure S9D. Histology at 180 days post-exposure. Higher magnification image of region D taken from R03007. Note the presence of edema (*) and mixed immune infiltration, predominatly lymphocytes (**), in the alveolar space. The trichrome section on the right is again from the same sample but several hundred microns away. Interstitial fibrosis is evident on both sections but highlighted readily by the blue staining of collagen deposition in the right section.

E

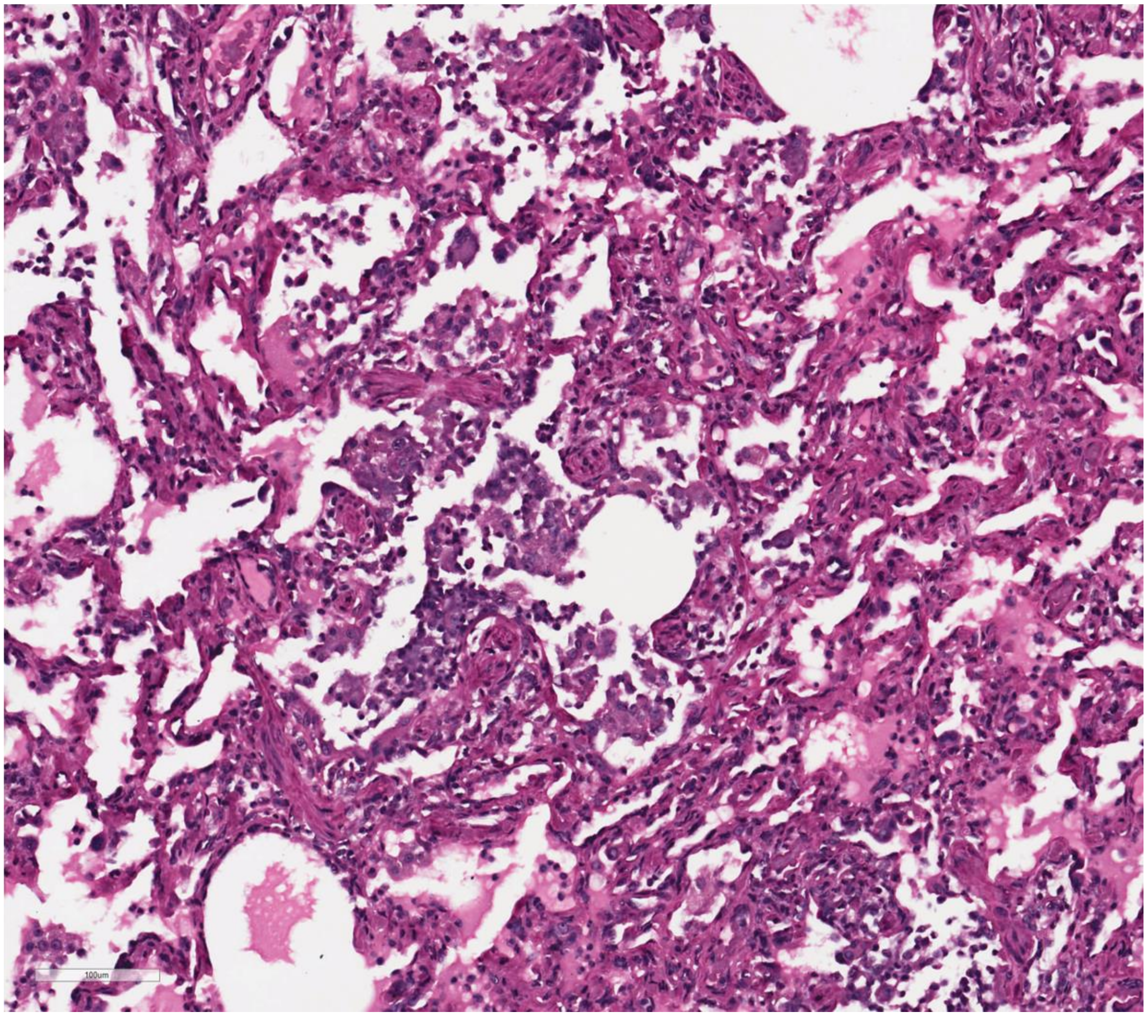

Figure S9E. Histology at 180 days post-exposure. Higher magnification image of region E taken from R03007.

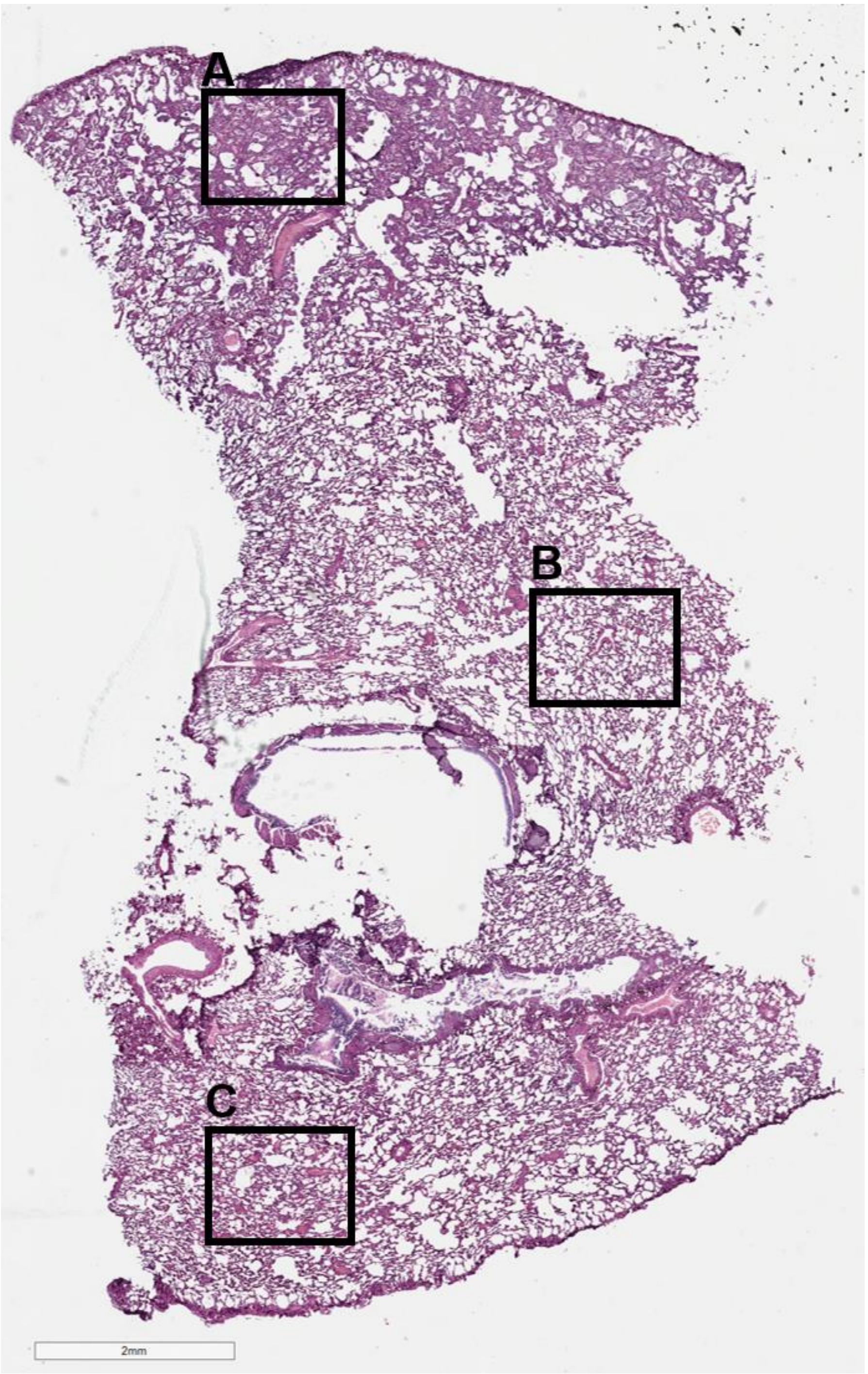

Figure S10. Histology at 180 days post-exposure. H&E stained section of irradiated animal, 040087. The boxes are representative regions of the pathologies observed in this animal at 180 days post-irradiation and are described below.

A

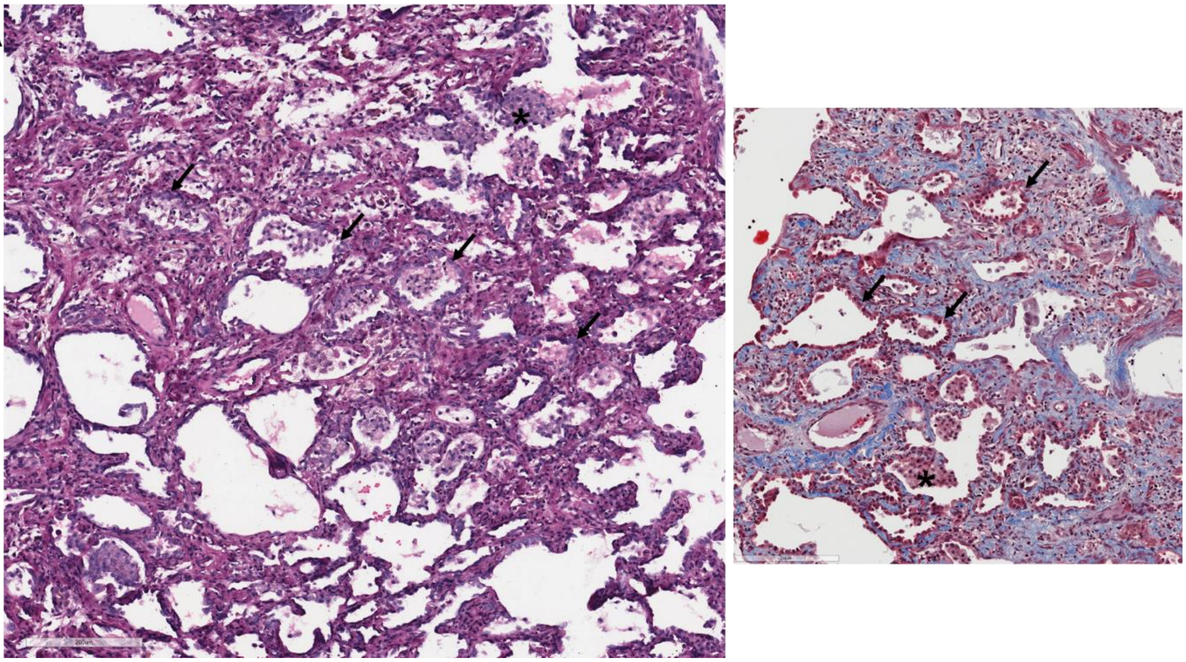

Figure S10A. Histology at 180 days post-exposure. Higher magnification image of region A taken from 040087. Note the accumulations of alveolar macrophages (*) within the alveolar lumen and alveolar and alveolar-bronchiolar hyperplasia (arrows). The thickened fibrotic alveolar walls are evident and further demonstrated by the blue collagen staining in the image on the right. This subpleural fibrosis is more dense and organized as evident by the darker blue collagen staining in the trichrome on the left. This trichrome section is from the same sample but taken several hundred microns from the sections used for MSI and its H&E section on the left.

B

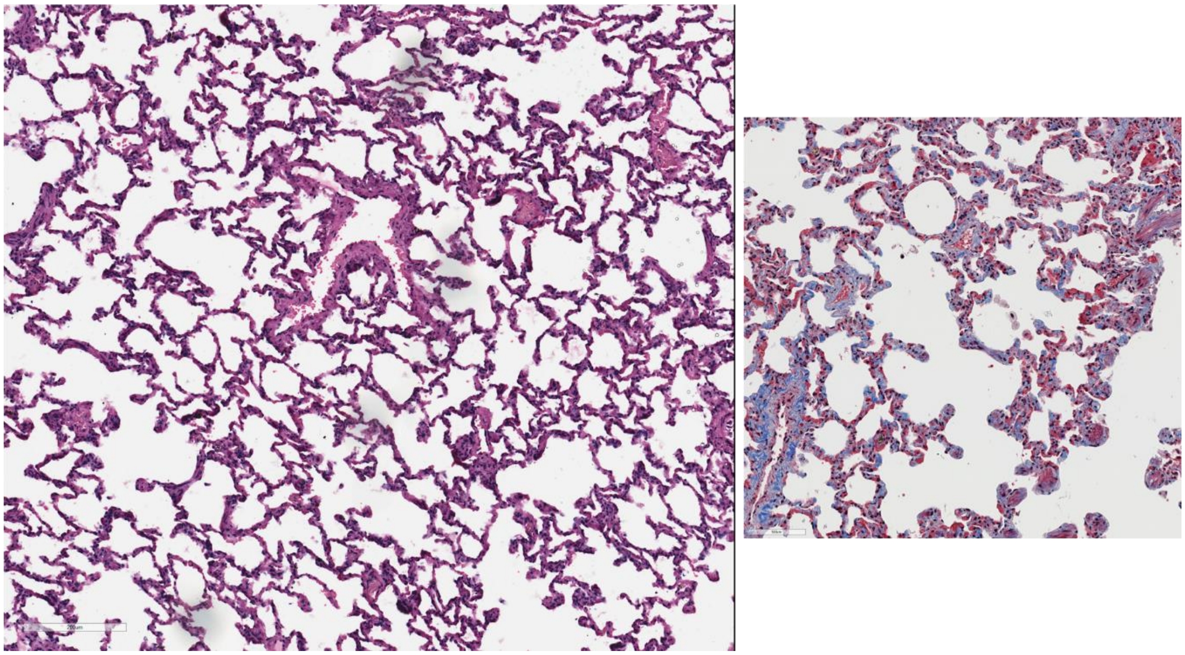

Figure S10 B. Histology at 180 days post-exposure. Higher magnification image of region B taken from 040087. The alveolar space is clear but there is evidence of thickening of the alveolar walls, which is further demonstrated by the light blue staining for collagen in the trichrome section on the right. This trichrome section is from the same sample but taken several hundred microns from the sections used for MSI and its H&E section on the left.

C

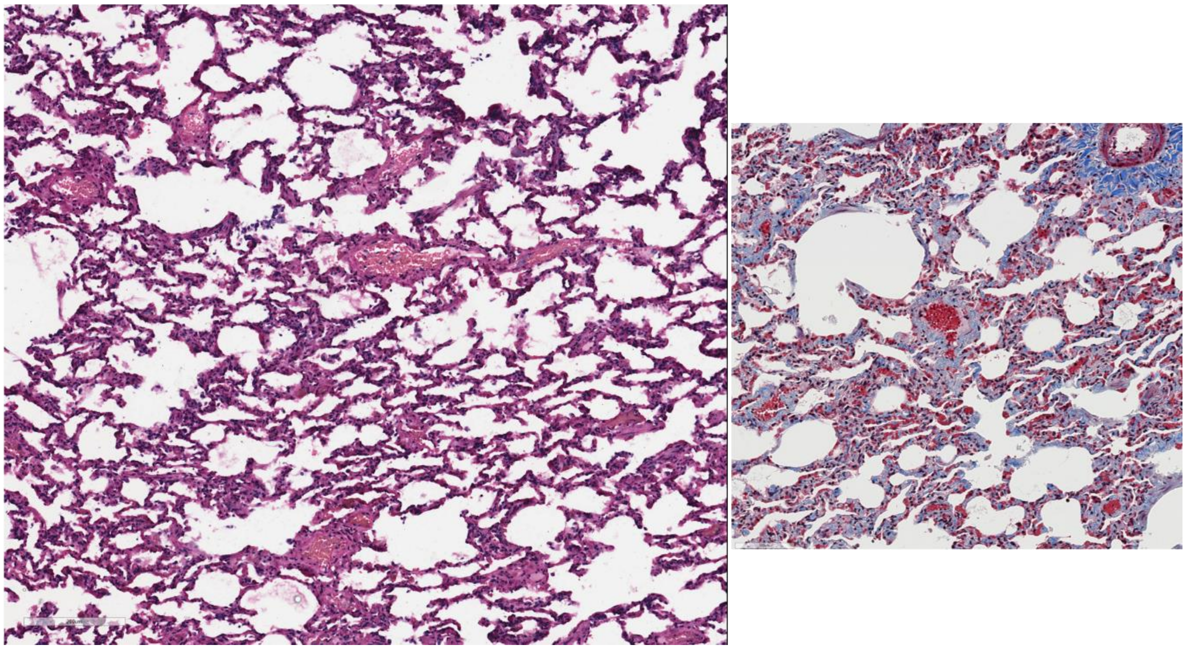

Figure S10 C. Histology at 180 days post-exposure. Higher magnification image of region C taken from 040087.

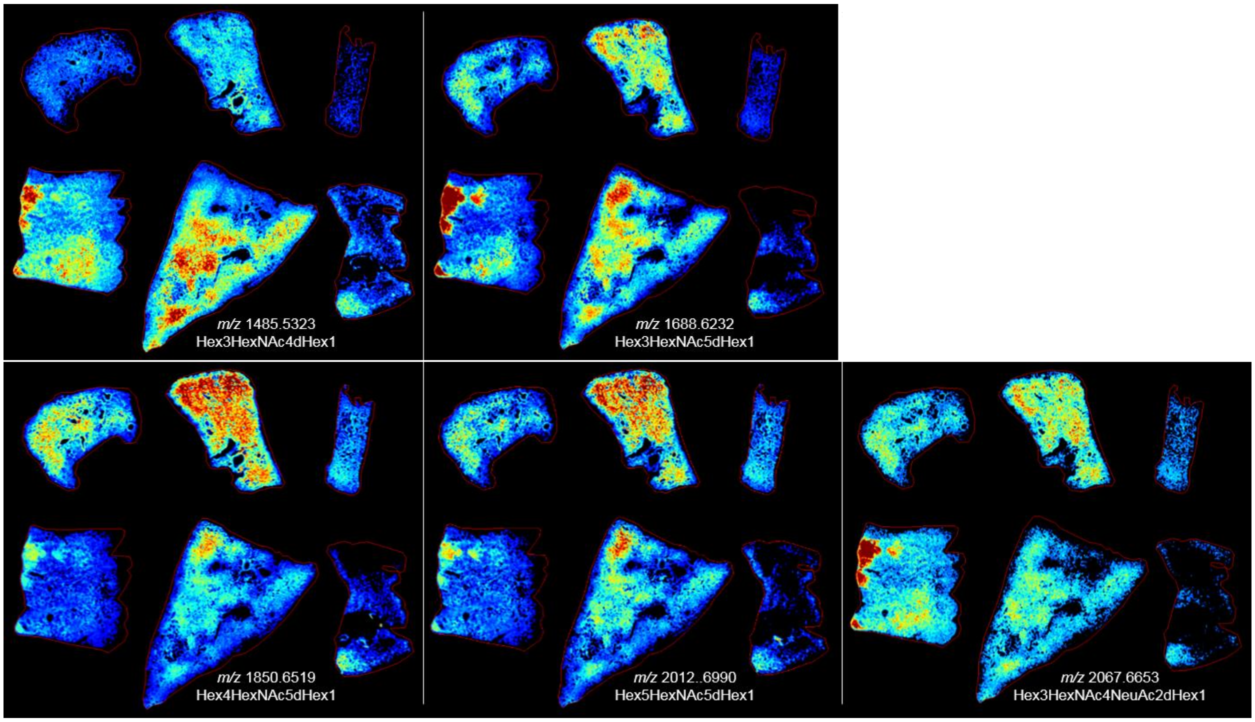

Figure S11. MSI at 180 days post-exposure. MALDI-MS images of N-glycans showing increases in regions of mucus accumulation in 04923 and regional increases in regions of R03007. Top, control: L-R, 0147, 0879, 08094011. Bottom, IR: L-R, 04923, R03007, 040087.

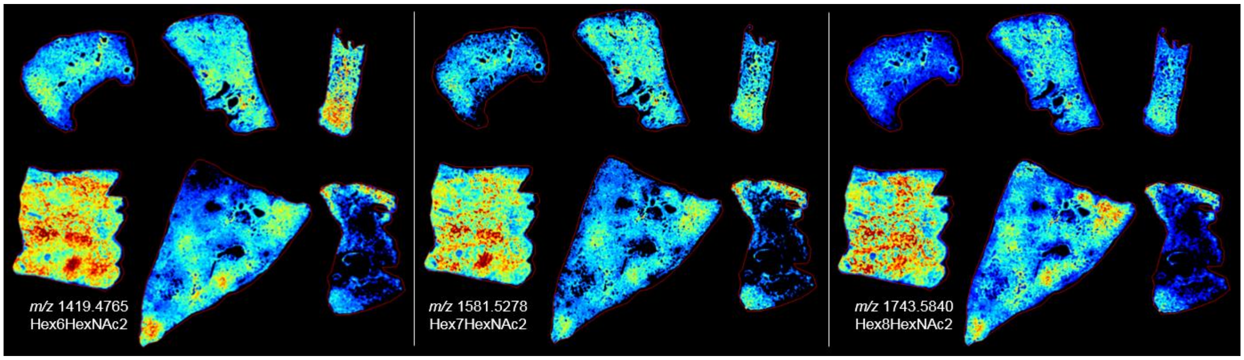

Figure S12. MSI at 180 days post-exposure. MALDI-MS images of high mannose N-glycans showing increases in regions of macrophage accumulation, AEC2 hyperplasia and alveolar-bronchiolar hyperplasia in 04923 and regional increases in regions of R03007. Top, control: L-R, 0147, 0879, 08094011. Bottom, IR: L-R, 04923, R03007, 040087.

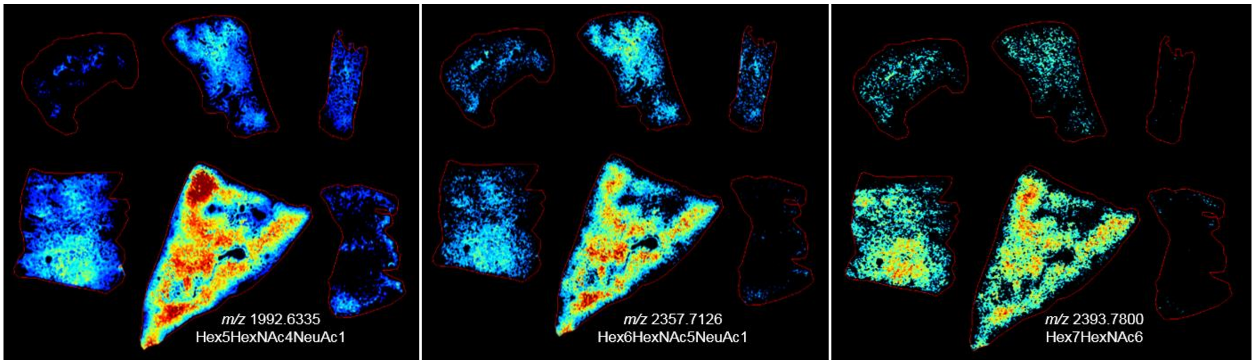

Figure S13. MSI at 180 days post-exposure. MALDI-MS images of N-glycans showing increases in regions of edema and mixed immune cell infiltration in sample R03007, with slight increases or localized increase in regions of macrophage accumulation in sample 04923. Top, control: L-R, 0147, 0879, 08094011. Bottom, IR: L-R, 04923, R03007, 040087.

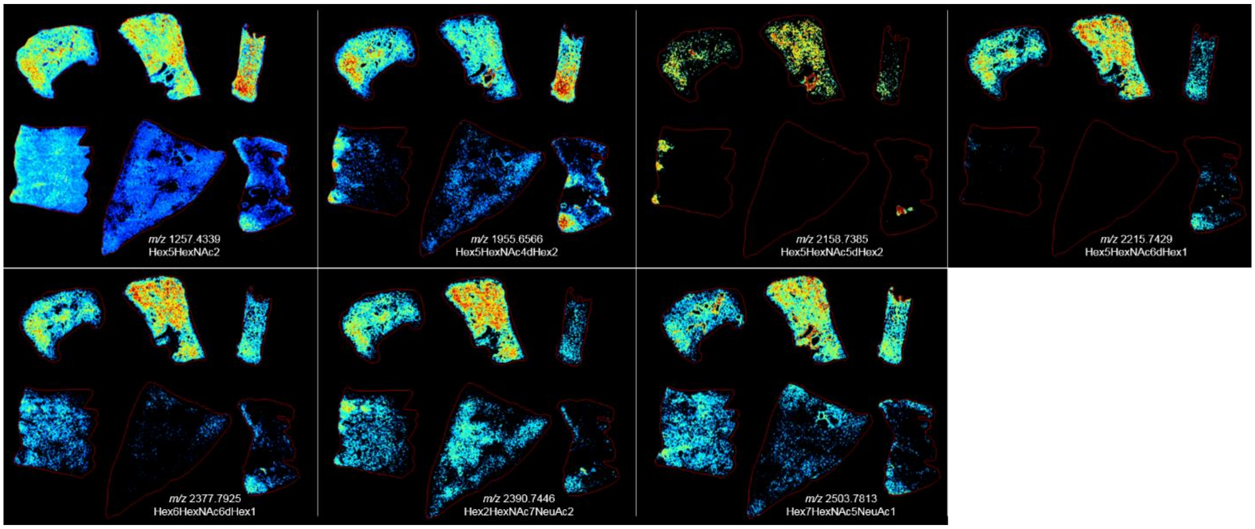

Figure S14. MSI at 180 days post-exposure. MALDI-MS images of N-glycans showing a decrease in the parenchyma of samples taken 180 days port-irradiation. Top, control: L-R, 0147, 0879, 08094011. Bottom, IR: L-R, 04923, R03007, 040087.

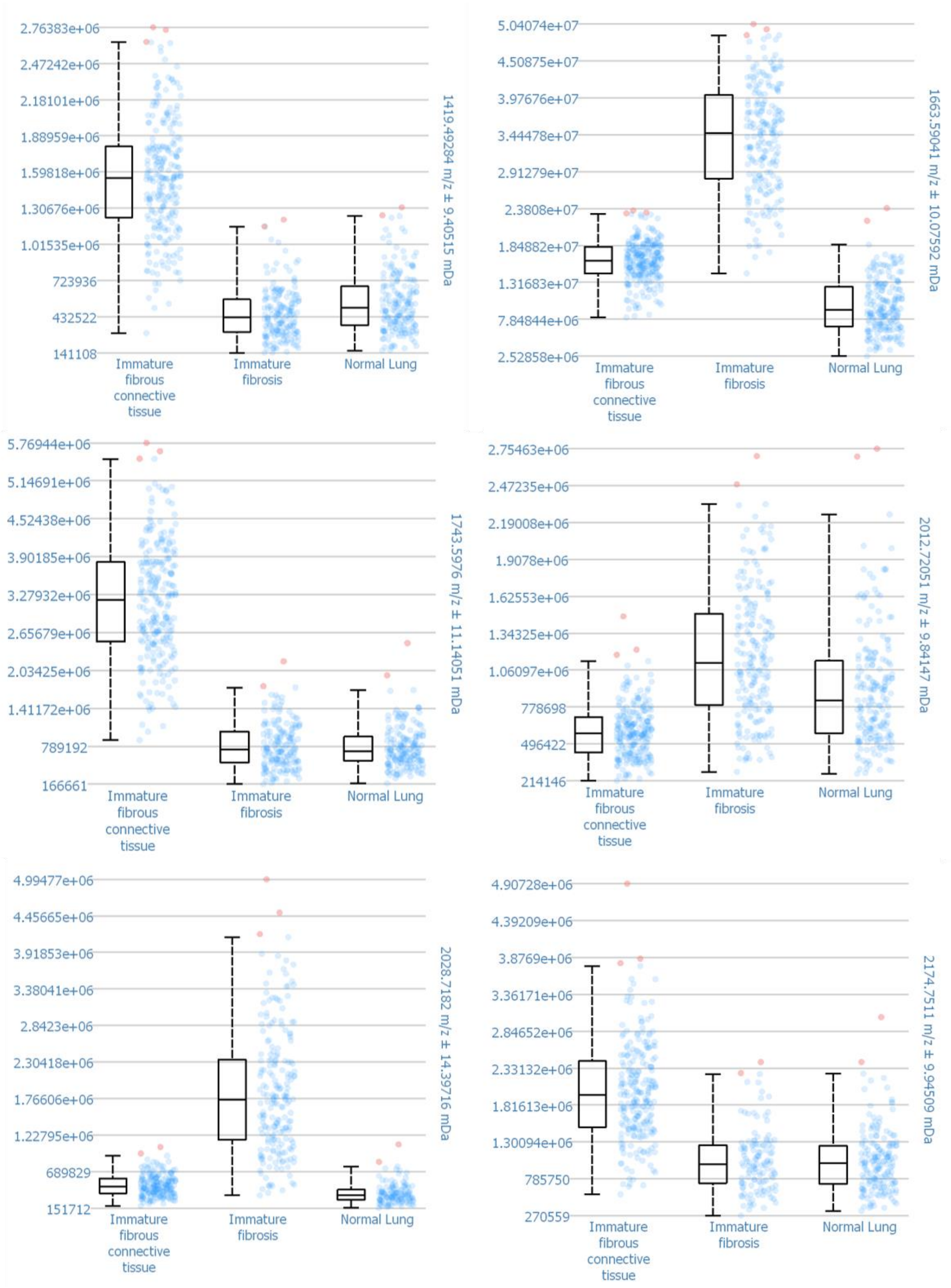

Figure S15. Boxplots, IR sample 040205 at 50 days post-exposure. Intensity box plot charts depicting the intensities of several glycans in the immature fibrous connective tissue region (left), the immature fibrosis region (middle) and the normal lung region (right) of sample 040205. Glycans shown are, Hex6HexNAc2 (top left), Hex5HexNAc4 (top right), Hex8HexNAc2 (middle left), Hex5dHex1HexNAc5 (middle right), Hex6HexNAc5 (bottom left) and Hex6dHex1HexNAc5 (bottom right).

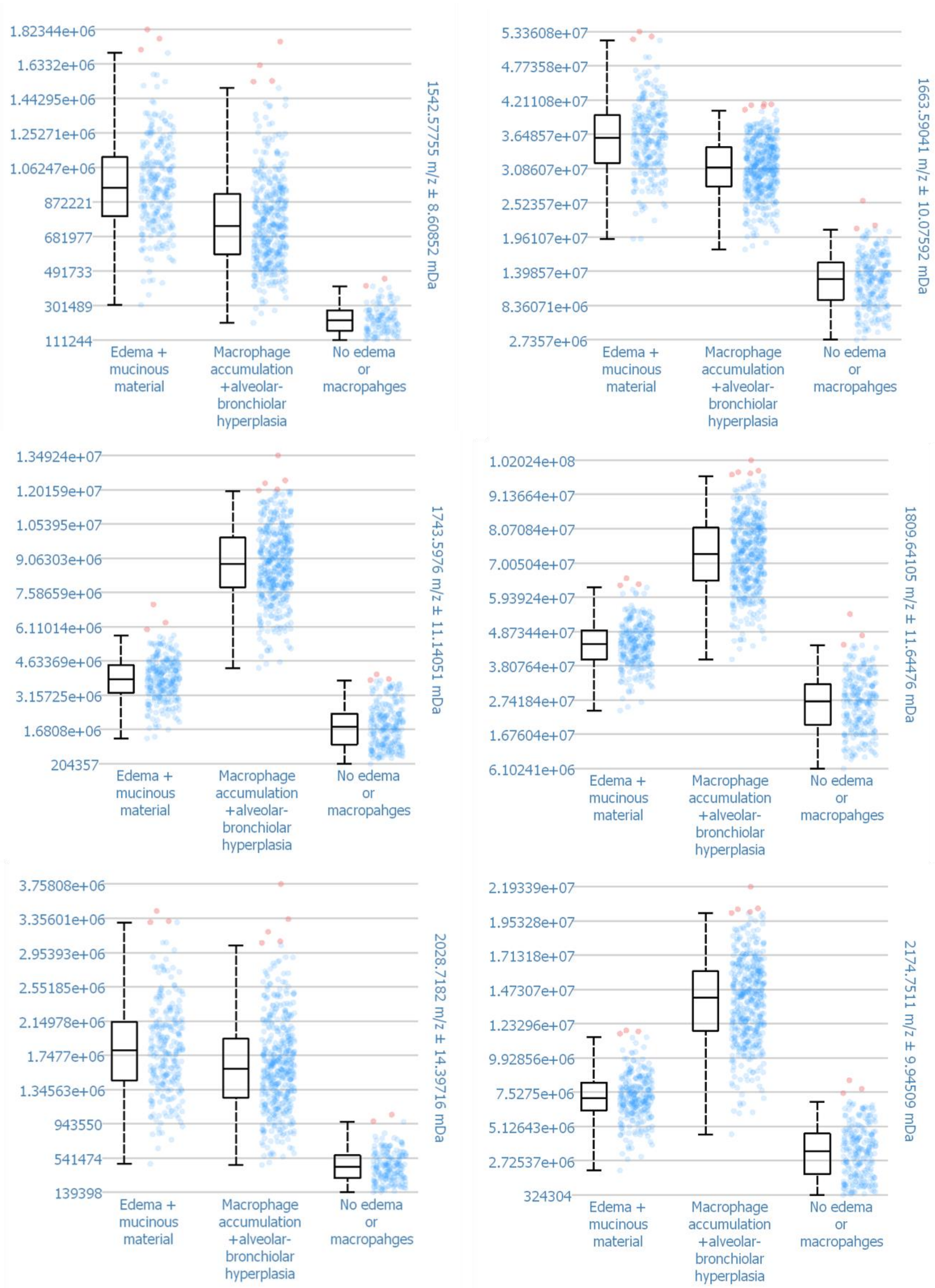

Figure S16. Boxplots, IR sample 061877 at 50 days post-exposure. Intensity box plot charts depicting the intensities of several glycans in the region containing edema and mucinous material (left), macrophage accumulation and alveolar-bronchiolar hyperplasia (middle) and regions with no edema of macrophages (right). Glycans shown are, Hex3HexNAc5 (top left), Hex5HexNAc4 (top right), Hex8HexNAc2 (middle left), Hex5HexNAc4dHex1 (middle right), Hex6HexNAc5 (bottom left) and Hex6dHex1HexNAc5 (bottom right).

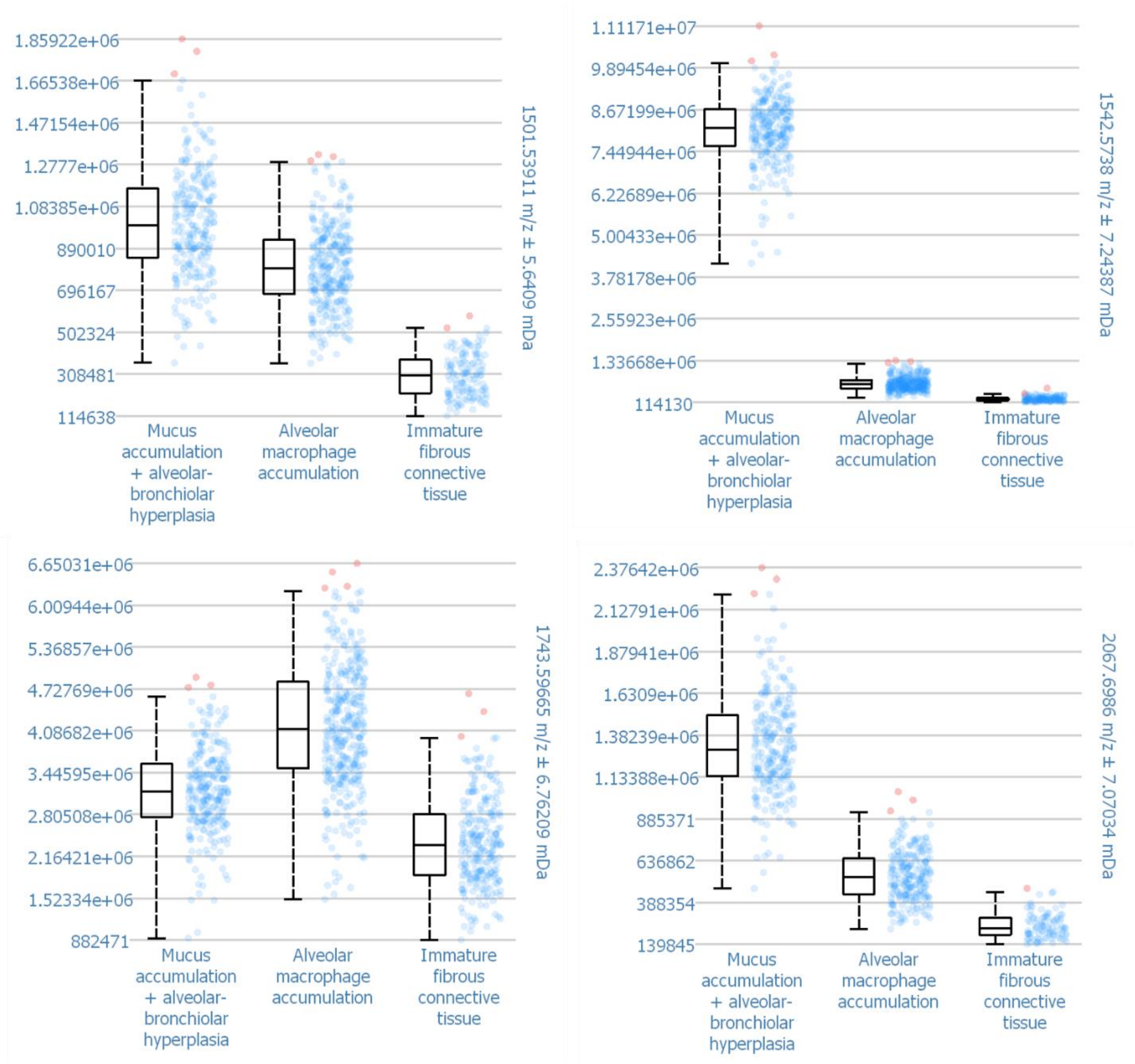

Figure S17. Boxplots, IR sample 04923 at 180 days post-exposure. Intensity box plot charts depicting the intensities of several glycans in the regions containing mucus accumulation and alveolar-bronchiolar hyperplasia (left), alveolar macrophage accumulation (middle) and immature fibrous connective tissue (right). Glycans shown are, Hex4HexNAc4 (top left), Hex3HexNAc5 (top right), Hex8HexNAc2 (bottom left) and Hex10HexNAc2 (bottom right).

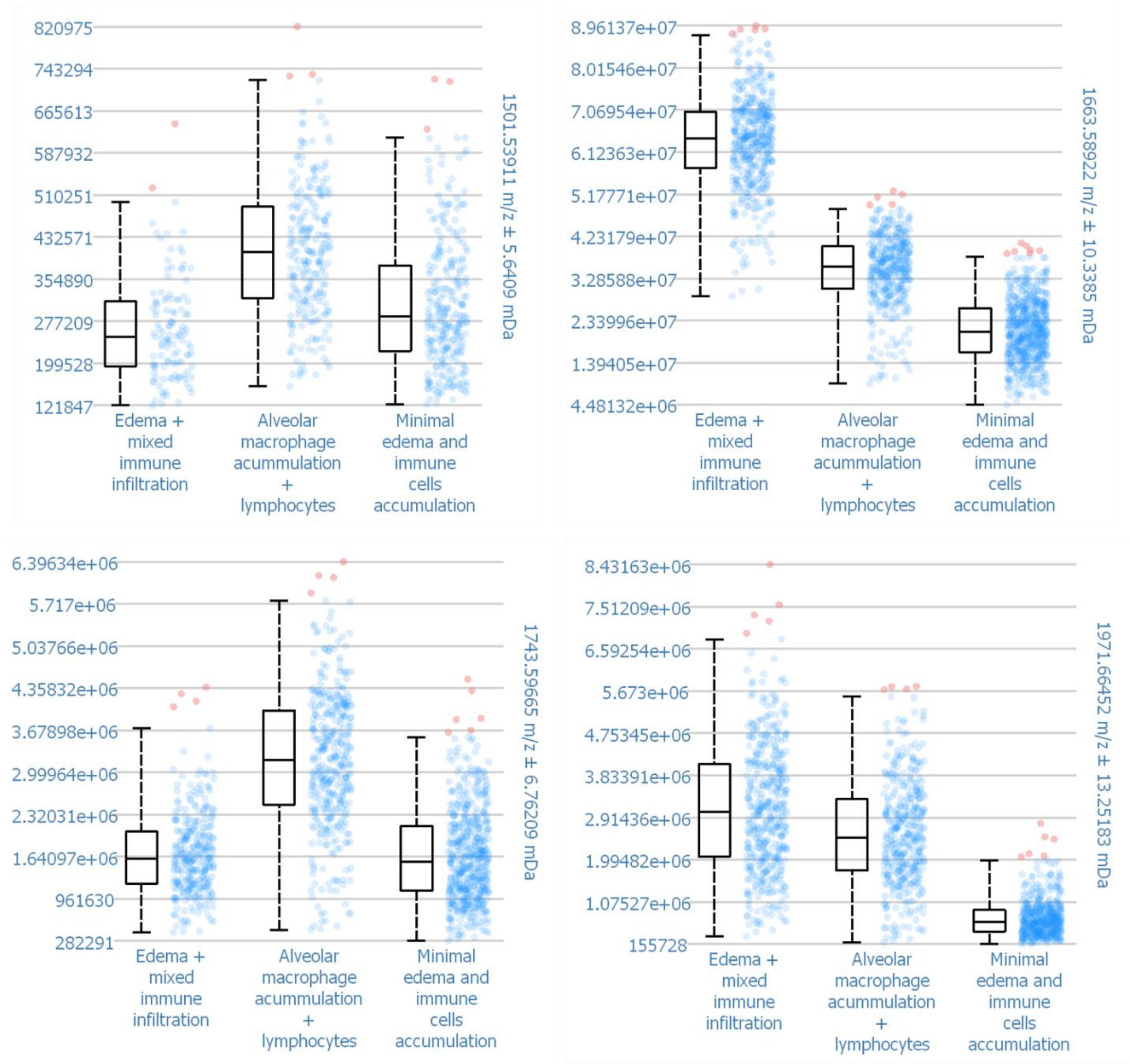

Figure S18. Boxplots, IR sample R03007 at 180 days post-exposure. Intensity box plot charts depicting the intensities of several glycans in the regions containing edema and mixed immune infiltration (left), alveolar macrophage and lymphocyte accumulation (middle) and regions with minimal edema and immune infiltrations (right). Glycans shown are, Hex4HexNAc4 (top left), Hex5HexNAc4 (top right), Hex8HexNAc2 (bottom left) and Hex4dHex1HexNAc4 (bottom right).
